# Parallels between stream and coastal water quality associated with groundwater discharge

**DOI:** 10.1371/journal.pone.0224513

**Published:** 2019-10-28

**Authors:** Tristan McKenzie, Henrietta Dulai, Jennet Chang

**Affiliations:** 1 Department of Earth Sciences, University of Hawai’i at Mānoa, School of Ocean and Earth Science and Technology, Honolulu, Hawai’i, United States of America; 2 College of Agriculture, Forestry, and Natural Resource Management, University of Hawai’i at Hilo, Hilo, Hawai’i, United States of America; University of Waikato, NEW ZEALAND

## Abstract

Groundwater-surface water interactions drive water quality in both streams and the coastal ocean, where groundwater discharge occurs in streams as baseflow and along the coastline as submarine groundwater discharge (SGD). Groundwater contributions to streams and to the coastal ocean were quantified in three urban streams in Kāne’ohe Watershed, Hawai’i. We used radon as a groundwater tracer to show that baseflow contributions to streams ranged from 22 to 68% along their reaches leading to the coast of Kāne’ohe Bay. Total SGD was 4,500, 18,000, and 23,000 m^3^/day for the northwest, central, and southern sectors of the bay, respectively. Total groundwater (stream baseflow + SGD) dissolved nutrient fluxes were significantly greater than those sourced from stream surface runoff. The studied streams exhibited increasing nutrient levels downstream from groundwater inputs with high nutrient concentrations, negatively impacting coastal water quality. SGD dynamics were also assessed during the anomalously high perigean spring tides in 2017, where SGD was four times greater during the perigean spring tide compared to a spring tide and resulted in strong shifts in N:P ratios, suggesting that rising sea level stands may disrupt primary productivity with greater frequency. This study demonstrates the importance of considering baseflow inputs to streams to coastal groundwater budgets and suggests that coastal water quality may be improved through management and reduction of groundwater contaminants.

## Introduction

Groundwater-surface water interactions impact nutrient and pollutant transport and directly affect water quality in streams and coastal ecosystems. Gaining reaches of streams receive groundwater, which affects stream discharge as well as its water quality [1]. Groundwater can also flow directly to the ocean as submarine groundwater discharge (SGD), and can be volumetrically comparable to stream discharge [2]. Polluted groundwater discharge to streams and coastline is a common problem for island watersheds with densely populated coastal plains, which in addition are often upstream of coral reefs and other sensitive coastal ecosystems that coastal communities depend on. Groundwater tends to be enriched in nutrients and other dissolved constituents sourced from land-use. For instance, non-channelized streams typically have nitrogen: phosphorus (N:P) ratios around 14, whereas N:P ratios in SGD commonly exceed the Redfield ratio of 16 [3–6]. Groundwater discharge can particularly impact streams in urban settings that may be fully or partially channelized, leading to a lack of hyporheic flow and riparian vegetation in addition to an increase in the velocity of stream water flow to the coastal ocean [7]. This study explores the evolution of groundwater and stream water quality in mostly channelized, gaining streams and the coastal ocean across a watershed and evaluates the role of groundwater on both stream and coastal water quality. Groundwater collects solutes from overlying land-use [4–6], meaning groundwater discharge directly affects surface water quality and should be of concern in stream and coastal water quality studies.

High volcanic pacific islands (HVPI), such as the Hawaiian Islands, are described by small watersheds that extend from the mountain ridge to the reef, steep topography, and permeable hydrogeology [8]. Fresh groundwater resources on HVPI are replenished from rainfall, and are stored in high-level aquifers confined by dike complexes, in basal lens aquifers, and less frequently in perched aquifers [8]. Groundwater from these aquifers can discharge either to streams that subsequently flow to the ocean or directly to the coastal ocean as SGD.

Streams are one vector of groundwater and groundwater-derived solute transport to the coastal ocean in Hawai’i. Perennial streams on the windward side of O’ahu, Hawai’i, are groundwater fed, with as much as 70% groundwater contribution to the total stream discharge in the form of baseflow during the dry season on average [9]. Streams tend to be prone to flash flooding and are fed by surface runoff particularly during the wet season [10]. Due to the steep topography and high-level dike impounded groundwater that is generally characteristic of windward Hawaiian watersheds, these streams are commonly gaining from dike complexes upstream (high-level aquifer baseflow), losing in mid-stream reaches, and gaining in the coastal plain from the basal aquifer (basal aquifer baseflow) [10]. These processes are collectively termed surface water-groundwater interactions and are known to drive stream water and chemical budgets [1]. While stream fluxes are volumetrically large, draining 48–69% of water output from the watershed [9], they still comprise by large part groundwater from baseflow.

Groundwater from coastal plain aquifer discharges to streams and estuaries and continuously along the coastline in the form of SGD. Although freshwater SGD is estimated to represent less than 10% of river discharge to the ocean globally [11–13], total SGD can be a major term in the water budget on a local scale [13–16]. On a local scale, SGD fluxes can comprise of up to two to four times greater water volumes compared to surface runoff, in addition to also transporting higher nutrient loads than surface pathways [14]. Globally, an estimated 2,400 km^3^/year of terrestrially derived fresh SGD is discharged, where major Pacific Islands, despite making up a comparatively small landmass, contribute to about 25% of global SGD [11]. Although both SGD and streams have been widely studied, few studies have looked at both comprehensively as a continuous system connected by subsurface hydrological pathways and the water quality trends along this continuum. This study shows that surface water quality is affected by groundwater discharge, which links streams and the coastal ocean. In other words, management actions eliminating contaminants from groundwater will be more effective than treating streams and coastlines as separate units.

The effect of groundwater on coastal water quality depends on the physical, biological and chemical processes [4, 17–18] that it undergoes once it discharges in the stream and its estuary or the subterranean estuary (STE) in the case of SGD. Analogous to a surface estuary, the STE connects terrestrially-derived groundwater and re-circulated seawater (both considered SGD) with the coastal ocean [4]. The STE is a subsurface zone that is highly biogeochemically active. Groundwater-derived dissolved nutrients undergo chemical transformations in the STE before entering the coastal ocean via SGD [4]. Most importantly, while there are changes in the hydraulic gradient between groundwater and the coastal ocean due to seasonality in precipitation as well as both semi-diurnal and semi-monthly tidal fluctuations [17–18], baseflow and SGD are usually persistent year round, whereas surface runoff tends to be associated with periods of high rainfall.

While groundwater affects multiple parameters of coastal water quality that are important from the perspective of coastal ecosystems (e.g. temperature, nutrient and heavy metal loads, salinity, alkalinity), nutrient loading has gained most attention for its linkage to eutrophication. Groundwater is comparatively nutrient-rich and generally exceeds the N:P Redfield ratio of 16:1 compared to the coastal ocean [4–6, 19] and SGD has been linked to coastal eutrophication, and harmful algal blooms from increased primary productivity [20–21] as well as decreased net community calcification [22–23].

Contaminants carried by groundwater are typically sourced from anthropogenic modifications to land-use (e.g. agricultural and industrial runoff; domestic and industrial wastewater), and can include substantial quantities of nutrients, heavy metals, and other regulated and unregulated chemicals. These may discharge to the coastal ocean either directly or indirectly via stream baseflow. Contaminants discharged to streams by baseflow may undergo biogeochemical transformations in the stream, during hyporheic exchange processes, or in the estuary [24]. Stream baseflow derived from the coastal basal aquifer, however, has a very short distance and stream travel time to the coast, meaning its composition, especially with respect to refractory chemicals, remains mostly unaltered. In addition, due to topography of coastal plains and estuarine hydrogeology, groundwater discharge is preferentially focused in estuaries compared to the coastal ocean [25]. Therefore, this study proposes that baseflow and SGD represent a continuum, i.e. there is no set boundary to where SGD and related contaminant flow ends, and rather than trying to define a boundary, it is just as important to look farther upstream in the watershed and evaluate high-level aquifer baseflow, basal aquifer baseflow, and SGD as different but dependent vectors of contamination to the coastal ocean. This allows for a better identification of the type and spatial extent of contaminant sources across the watershed. From a management perspective, characterizing groundwater quality and discharge locations may explain the sources of many stream and coastal water quality problems.

In that context, this study examines surface and groundwater interactions, with a main focus on groundwater discharge, along a continuum from the upstream reaches of streams to the coastal ocean, i.e. a ridge to reef extent. The study area is in Kāne’ohe Bay, O’ahu, Hawai’i where groundwater flow as well as surface runoff have been identified as sources of persistent stream and coastal water contamination [16, 26–27]. Surface and groundwater contributions to water discharge across the stream-coastline continuum are significant for (1) water budgets of streams and the coastline, and (2) fractions of dissolved load contributions to overall water quality in both streams and the coastal ocean. While the former is important to know for water budgets and resources management, it is also the basis for our understanding and management of the latter. An additional dimension to this complex problem is sea level rise, coastal flooding and extreme tides that affect the fresh and saline components of baseflow and SGD.

In the summer of 2017, Hawai’i experienced anomalously high perigean spring tides (or “king tides”) with a tidal range up to 1.03 m (June 23, 2017) compared to the average range of 0.45 m [28]. These anomalously high tides caused localized flooding, both surface flooding sourced directly from the high tidal height as well as indirectly via groundwater inundation [28]. This study captured SGD during the perigean spring tides and compares SGD and its composition to regular tidal events. This natural experiment gives us a peek into the future on how SGD and solute fluxes will be different at a future higher sea level stand.

## Materials and methods

### Study site

**Fig 1:**
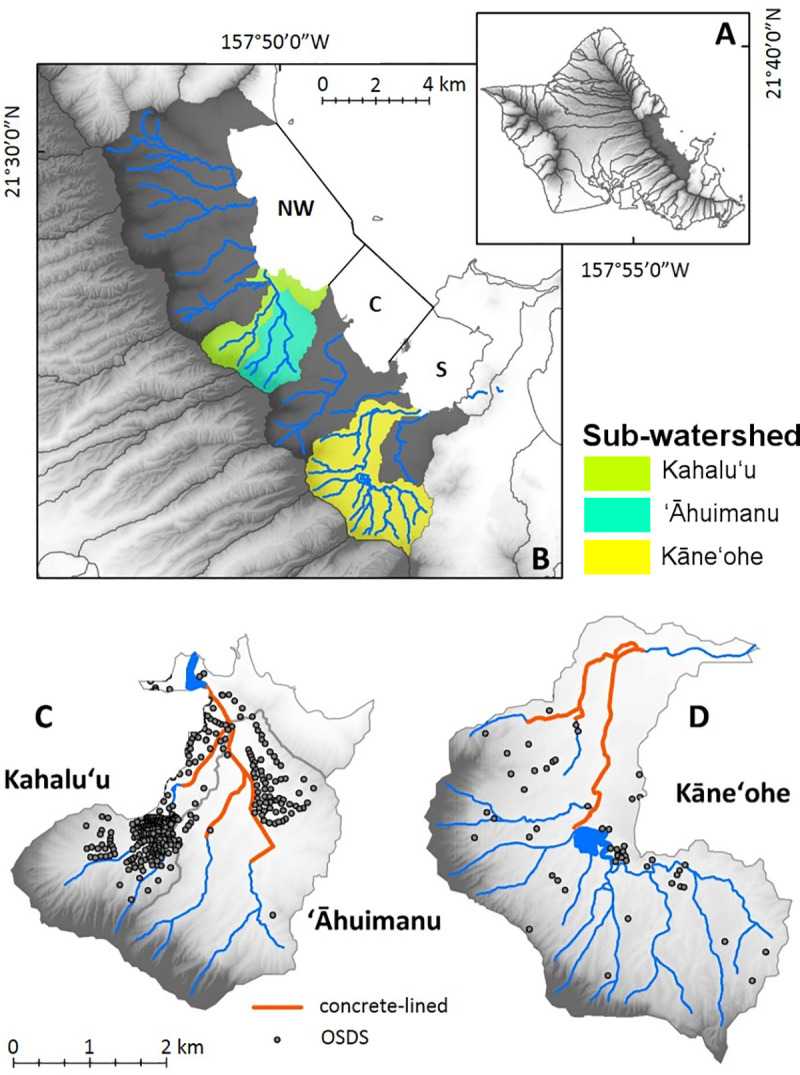
Map of study area. (A) Location of study area on the island of O’ahu, (B) Sectors of Kāne’ohe Bay (NW, C, and S), Kāne’ohe watershed and streams, and sub-watersheds (Kahalu’u, ’āhuimanu, and Kāne’ohe) studied. Detailed view of (C) Kahalu’u and ’āhuimanu and (D) Kāne’ohe sub-watersheds. Portions of the stream that are lined with concrete are in orange. Each dot represents an individual onsite sewage disposal system (OSDS), which are predominantly cesspools in the region [32].

The study was conducted in three sub-watersheds of Kāne’ohe Watershed (Kahalu’u, ’āhuimanu, and Kāne’ohe) feeding into Kāne’ohe Bay, O’ahu, Hawai’i and the bay’s nearshore waters (Fig 1). Kāne’ohe Bay is subdivided into three sectors (northwest, central and south), which differ in terms of residence time, bathymetry, and influence from land-use. The larger Kāne’ohe Watershed has seven perennial streams that feed into Kāne’ohe Bay and is partitioned into fourteen steep amphitheater-shaped sub-watersheds (Fig 1; [29–30]). An estimated 96 million m^3^/year of freshwater enters Kāne’ohe Bay [31].

#### Geology

**Fig 2:**
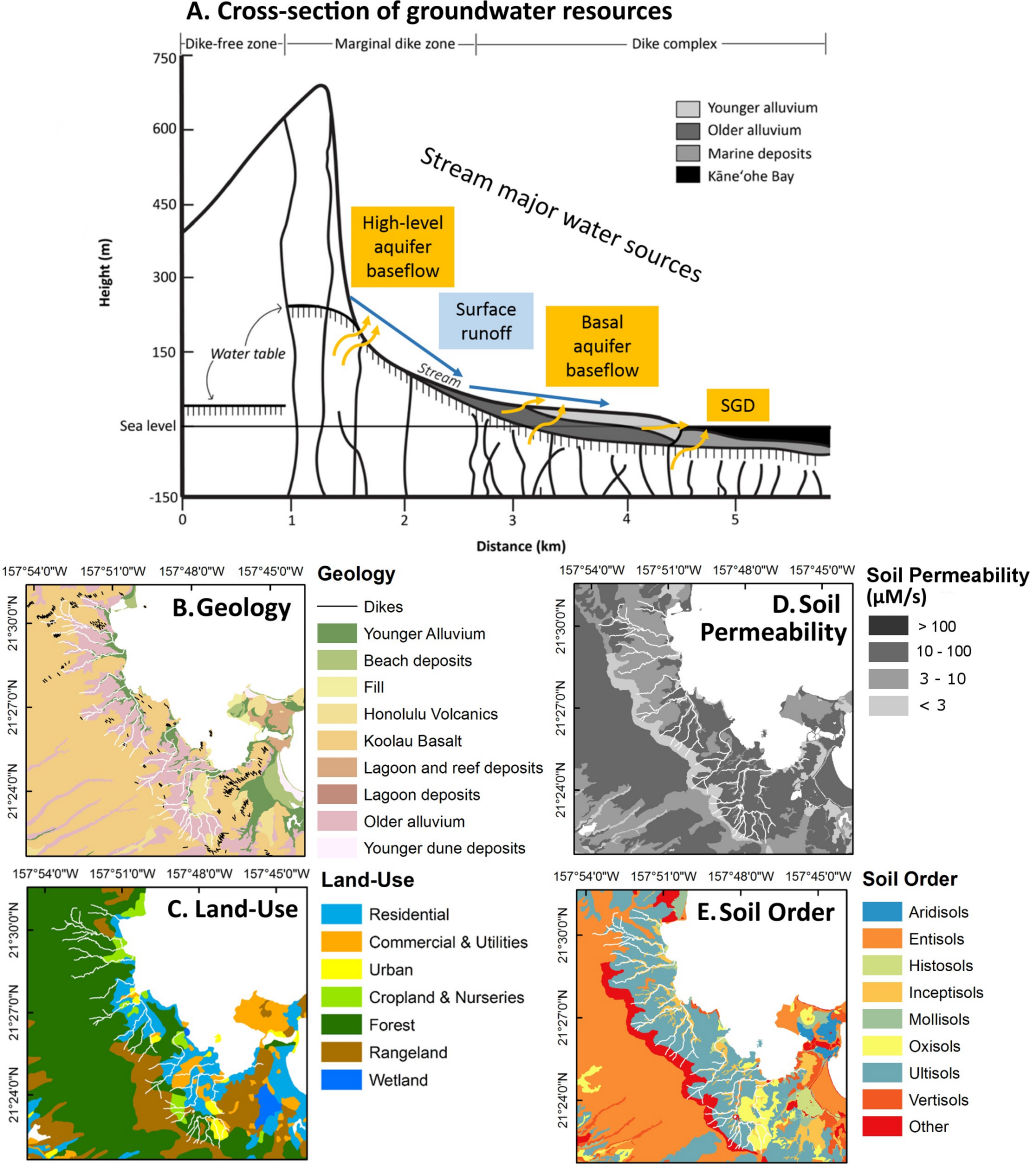
Geology of Kāne’ohe Watershed and Bay. (A) Idealized cross-section of groundwater resources and flow in Kāne’ohe Watershed and Bay (after [31]); (B) geology; (C) land-use; (D) soil permeability, and (E) soil order of the study area [36, 37, 38].

Kāne’ohe Watershed is mostly comprised of basalt with overlying alluvium. Ko’olau basalt (theoleiitic in composition) is the prevailing basalt type in Kāne’ohe watershed, and its thickness ranges from 0.6 to 24 m (3 m on average) (Fig 2; [8, 33–34]). The younger Honolulu volcanic series are interspersed throughout the watershed and are generally of an alkalic composition. Alluvium (sand, silt, clay, and gravel) covers about 60% of the coastal plains in the watershed [35]. Soils in the study area are predominantly utisols (kaolinite-rich, high capacity for phosphorus fixation), oxisols (rich in oxide-clay minerals, high capacity for phosphorus fixation), and inceptisols (Fig 2; [36]).

#### Kāne’ohe Bay and watershed

Kāne’ohe Bay is a reef-dominated embayment located on the windward side of O’ahu and has been historically, as well as currently, susceptible to contamination [27, 30, 39–40]. From 1963 until 1979, municipal sewage effluent was directly discharged to the southern portion of Kāne’ohe Bay, resulting in low oxygen conditions, high primary productivity in the water column, and coral reef areal decline [29–30, 39]. After the elimination of the sewage effluent outfall, surface runoff has been thought to be the major pathway responsible for delivering contaminants to Kāne’ohe Bay [27, 41], recent research has indicated that SGD-derived nutrient inputs [16] are comparable to those coming from surface runoff [30].

The windward slopes of the Hawaiian Islands receive high quantities of rainfall due to orographic lifting and prevailing trade-wind patterns [42]. Precipitation tends to be relatively consistent in the upper slopes however, rainfall on O’ahu’s coastal plains occurs mostly (about 70% of annual rainfall) from October through April [35]. Seasonality between surface runoff and groundwater discharge dominance into Kāne’ohe Bay are anticipated due to high rainfall during the wet season (Table 1). During the wet season, surface runoff is the dominant input of freshwater into the bay [43]. Groundwater storage, however, is not instantaneously discharged and thus dominates freshwater flow during the dry season, peaks about 4 to 5 months later [43]. The annual average water budget of the watershed can be broken down to 2400 mm precipitation, 1350 mm evapotranspiration, 800 mm recharge, and 350 mm surface runoff, or about 56%, 33%, and 11% of total precipitation, respectively [42, 44].

**Table 1:** Comparison of Kāne’ohe Bay’s watersheds by sector: northwest (NW), central (C), and southern (S).

Sector	Area	Total Annual Precip	Dry Season Precip	Wet Season Precip	Avg. Annual Precip	Stream Length	Stream Q	Base flow	Recharge from OSDS*
	*km* ^*2*^	*mm*	*mm*	*mm*	*mm*	*km*	*%*	*10*^*4*^ *m*^*3*^*/d*	*10*^*4*^ *m*^*3*^*/d*
NW	31.4	3140	2010	1130	2380	46.1	50	3.93	0.325
C	27.0	2410	1404	1002	1900	27.2	25	1.62	
S	32.5	2660	1720	938	2130	38.9	25	3.04	0.0988

Watershed area is from [45]. Rainfall for the 2016 dry season (May–October 2016) and wet season (November 2016 –April 2017) [46], missing values were interpolated using 30-year average monthly values [42]. Total stream length and percentage of discharge of total stream input into Kāne’ohe Bay are from [26, 45, 47]. Stream baseflow is estimated to be 70% of daily mean stream flow [26, 45, 48]. Recharge from OSDS are based on estimates from [32].

* Northwest and central sectors were considered as one.

The three sub-watersheds and streams studied represent a large variability in human development and population density and were selected to highlight land-use differences. The Kahalu’u and ’āhuimanu sub-watersheds drain into north-central Kāne’ohe Bay. Kahalu’u Stream flows into ’āhuimanu Stream about 250 m before discharging into Kahalu’u Estuary (Fig 1). Additionally, downstream portions of both streams are channelized in concrete-lined culverts for about 1.6 km prior to feeding into Kahalu’u Estuary. Kahalu’u has comparatively lower population and area than ’āhuimanu, but has a significantly higher OSDS density and number of cesspools (Tables 2 and 3) because the sewer connection only serves ’āhuimanu. Other potential sources of contaminants are sourced from agriculture [45]. The Kāne’ohe sub-watershed drains into southern Kāne’ohe Bay. Upstream reaches of Kāne’ohe Stream are predominantly undeveloped compared to downstream reaches and the stream has two main tributaries, Kamo’oali’i and Kapunahala. Kāne’ohe Stream is intermittently channelized for 4 km in concrete culverts. Kāne’ohe sub-watershed has substantially fewer OSDS, however it is comparatively more urban compared to the other studied areas. Agriculture is another potential contaminant source in Kāne’ohe [45].

**Table 2:** Comparison of the studied sub-watersheds.

Sub-watershed	Area	Max Elev.	Population	Impervious surface	OSDS density	Cesspool	GW withdrawal by pumping
	*km* ^*2*^	*m*		*%*	*units/ km* ^*2*^	*% (# of units)*	*10*^*4*^ *m*^*3*^*/d*
Kahalu’u	3.38	768	4,738	13.1	33.1	76 (234)	4.5
’āhuimanu	6.24	859	8,810	1.21	11.2	74 (82)	
Kāne’ohe	14.7	851	34,597	22.8	3.73	91 (50)	2.2

Area and maximum elevation are from [45]. Population is based off the 2010 United States Census. Percent impervious surface represents the area of the sub-watershed, which has been developed in a way that prevents water infiltration [45]. OSDS density includes the number of cesspools, septic tanks, aerobic, and soil treatment units divided by the area of the sub-watershed [32]. Percent cesspool represents the number of cesspools compared to the total OSDS in each sub-watershed, and the number of cesspool units [32]. Groundwater withdrawal by pumping rates are from [45].

**Table 3:** Comparison of the studied streams.

Stream	Stream Length	Stream lined with concrete	Wet Season Q	Dry Season Q
	*km*	*%*	*10*^*4*^ *m*^*3*^*/d*	*10*^*4*^ *m*^*3*^*/d*
Kahalu’u	3.53	35	1.13	0.821
’āhuimanu	5.34	56	2.78*	2.03*
Kāne’ohe	10.9	37	4.00	2.81

Total stream length is calculated from a GIS layer [49]. Percentage concrete refers to the percentage of the total stream length that has been altered and lined with concrete (as opposed to natural substrate). Wet and dry season discharge from USGS Stream Gages [48].

* indicate there is no active USGS stream gage present and stream discharge was estimated based off of relative discharge between Kahalu’u and ’āhuimanu Streams established in previous literature [26, 45]. Kāne’ohe Stream includes Kamo’oali’i and Kapunahala tributaries and upper Kāne’ohe stream.

### Sample collection and analysis

Our goals were to quantify (1) ground and surface water fluxes within the watershed along streams and the coastal ocean and (2) to characterize water quality in ground and surface water fractions through basic water quality parameters (temperature, conductivity and dissolved oxygen) as well as dissolved nutrient concentrations. Surface water was sampled from the coastal zone and streams, and groundwater was collected from the beach face (at depths ranging from 20 to 60 cm), stream bank weepholes, and upland wells through a series of snapshot studies aiming to capture both dry and wet seasons between September 2016 through July 2017 for Kahalu’u and ’āhuimanu sub-watersheds and July through November 2017 for Kāne’ohe sub-watershed. Land access was granted through the Hawai’i Department of Transportation Windward Baseyard and the Honolulu Board of Water Supply. Groundwater samples were taken from locations with visible groundwater discharge. Coastal water surveys for Kāne’ohe Bay were conducted only during the dry season. In addition, high spatial resolution studies were carried out along the coastline and in the streams feeding the northwestern (Kahalu’u and ’āhuimanu Streams) and southern (Kāne’ohe Stream) sectors of Kāne’ohe Bay in order to gain a better understanding of the role of groundwater along the stream-coastal ocean continuum. Groundwater in stream banks and along the shoreline were collected with a peristaltic pump though push-point samplers (MHE Products). Total stream discharge rates were higher during the dry season compared to the wet season during our study period due to dry season fieldwork concurring with La Niña conditions (Ocean Niño Index (ONI): -0.7 ± 0.5°C) known to cause wetter dry seasons, which was subsequently followed up by an atypically dry wet season [50]. Because of the co-occurrence of fieldwork with La Niña, we will subsequently refer to the dry (May through October) and wet (November through April) seasons as “July” and “February” sampling periods, respectively.

#### Water fluxes

Stream discharge was measured in regular intervals along the streams to determine both total flow and gaining portions via seepage runs [51] using a stream flow meter (SonTek Flowtracker). Since this method may not capture simultaneous in and outflow, groundwater discharge was also estimated using a ^222^Rn (radon) mass balance (see section 3.3 below) for which radon measurements were performed in the stream and along the coastline. Ground (n = 76) and surface water (n = 97) radon grab samples were collected into 250 mL glass bottles and analyzed the same day with a RAD-H_2_O radon-in-air analyzer equipped with water analysis accessory (Durridge Inc.). Measured radon activities were decay-corrected to the time of sample collection. The maximum radon groundwater radon concentration from each sector or stream (n = 6) was used as an end-member for the radon mass balance models described below. We used maximum concentrations because they provide the most conservative SGD estimates because other processes such as tidal pumping may factor into our estimates.

In addition to grab sampling, surface water surveys along the coastline and in streams were conducted using a RAD-AQUA (Durridge Inc.) placed into a wheel barrel or kayak. For tidally influenced locations, radon surveys were conducted at low tide, when SGD is predicted to be highest [52]. This was achieved by continuously pumping water with a bilge pump through an air-water exchanger and then into the radon-in-air analyzer with a measurement interval of five minutes. Measurements of conductivity, temperature, and depth were taken simultaneously with a CTD probe (both a Schlumberger Inc. CTD diver and YSI Multiparameter Sonde (V2-2 6960) were used) to allow for correction of radon inventories and to calculate a radon mass balance [52, 53].

Three radon time series were conducted in Kahalu’u Estuary and Beach between May and June 2017. Two were conducted during the 2017 perigean spring tide at Kahalu’u Estuary (21.4570, -157.8385) and Kahalu’u Beach Park (21.4602, -157.8398) during the May 2017 perigean spring tide (tidal range = 0.90 m), and June 2017 perigean spring tide (tidal range = 0.99 m), respectively. The third time series was done at the same location at Kahalu’u Beach Park, during a spring tide (tidal range = 0.66 m).

#### Water quality

Ground and surface water were sampled for dissolved nutrients. Water quality parameters such as temperature and salinity were measured with an YSI Multiparameter Sonde (V2-2 6960). Dissolved nutrient samples were filtered upon collection through a 0.45 μm filter into acid-cleaned 60 mL HDPE bottles and stored in dark and at 4°C until analysis. Samples were analyzed for Total Dissolved Nitrogen (TN), Total Dissolved Phosphorus (TP), NO_3_^-^ + NO_2_^-^ (because of negligible NO_2_^-^, from here on only listed as NO_3_^-^), PO_4_^3-^ (DIP), NH_4_^+^, and SiO_4_^4-^ (DSi) with a SEAL AutoAnalyzer 3 HR in the S-Lab at the University of Hawai’i, Mānoa. One in every ten samples were analyzed in duplicate for quality control and to estimate measurement uncertainties for each batch of measurement. Sample precisions within one standard deviation based on duplicates were 0.10 μM for NO_3_^-^, 0.19 μM for NH_4_^+^, 0.015 μM for PO_4_^3-^, and 2.5 μM for DSi. Dissolved inorganic nitrogen (DIN) concentrations were calculated as the sum of NO_3_^-^ and NH_4_^+^, and DON concentrations were determined by difference between TN and DIN.

Nutrient concentrations were corrected for salinity using previously established coastal end-members from Kāne’ohe Bay (Table 4).

**Table 4:** Dissolved nutrient coastal and ridge end-members.

		Nutrient concentrations (μM)			
	Salinity	DIN	DIP	DSi	DON
Coastal	35	1.9	0.29	36	4.7
Groundwater	0.076 ± 0.021	12 ± 2.7	1.5 ± 0.41	530 ± 88	2.8 ± 2.7

Coastal nutrient (DIN and DIP) end-members are from [29], DSi bay end-member from [54], and DON end-member from [30]. Groundwater end-members (n = 10) are the mean values from upland wells sampled in this study, which span the three studied sectors of the watershed.

The coastal end-members were used to correct dissolved nutrient concentrations for salinity with Eq 1 where C* represents the salinity corrected concentration, C_mix_ is the uncorrected sample concentration, C_b_ is the bay end-member concentration, S_mix_ is the salinity of the sample, S_gr_ is the salinity of the groundwater end-member, and S_b_ is the salinity of the bay end-member.


$$C*=Cmix+(Cmix-Cb)\times \frac{\left(Smix-Sgr\right)}{\left(Sb-Smix\right)}$$ (1)

Nutrients were corrected for salinity with the assumption that nutrient concentrations in excess of the coastal end-member are terrestrially sourced and thus to allow for estimation of land-derived nutrient fluxes, where brackish and saline samples are diluted by salty bay water.

#### Groundwater and nutrient flux calculation

Radon mass balances derived from [52–53, 55] were calculated for both riverine and coastal settings resulting in groundwater fluxes. Total SGD fluxes in m^3^d^-1^ (includes both fresh and re-circulated saline SGD) along the coastline were calculated using Eq 2, where A_Rn_sw_ and A_Rn_gw_ are the coastal ^222^Rn activities, corrected for in-situ ^222^Rn produced by ^226^Ra and by diffusion from sediments as well as losses due to atmospheric evasion [56] (both in (Bq m^2^ day^-1^), and groundwater ^222^Rn end-member stream and sector of the bay, V is the volume of water represented by the length of shoreline per measurement, water depth and distance from shore (m^3^), and *τ* is the coastal residence time of the water (we conservatively used 12.42 hours, reflecting flushing by semi-diurnal tides, acknowledging that certain areas may have faster circulation).


$${QSGD}_{tot}=\frac{A_{Rn\_sw}\times V}{\tau \times A_{Rn\_gw}}$$ (2)

Sources of uncertainty in SGD estimation arise from the choice of ^222^Rn end-member, water residence time, and assumptions of static conditions (i.e. no spatiotemporal variation) per volume of water used in the mass balance. Uncertainties associated with these parameters are propagated throughout the calculation of SGD. Gas transfer velocities calculated using wind speed [56] were in agreement with those found using ^3^He/SF_6_ in Kāne’ohe Bay [57]. Fresh and saline SGD fluxes were estimated using Eq 3, after [52].


$${QSGD}_{fresh}=\frac{(S_{b}-S_{sample})\times V}{\tau \times S_{b}}$$ (3)

**Fig 3:**
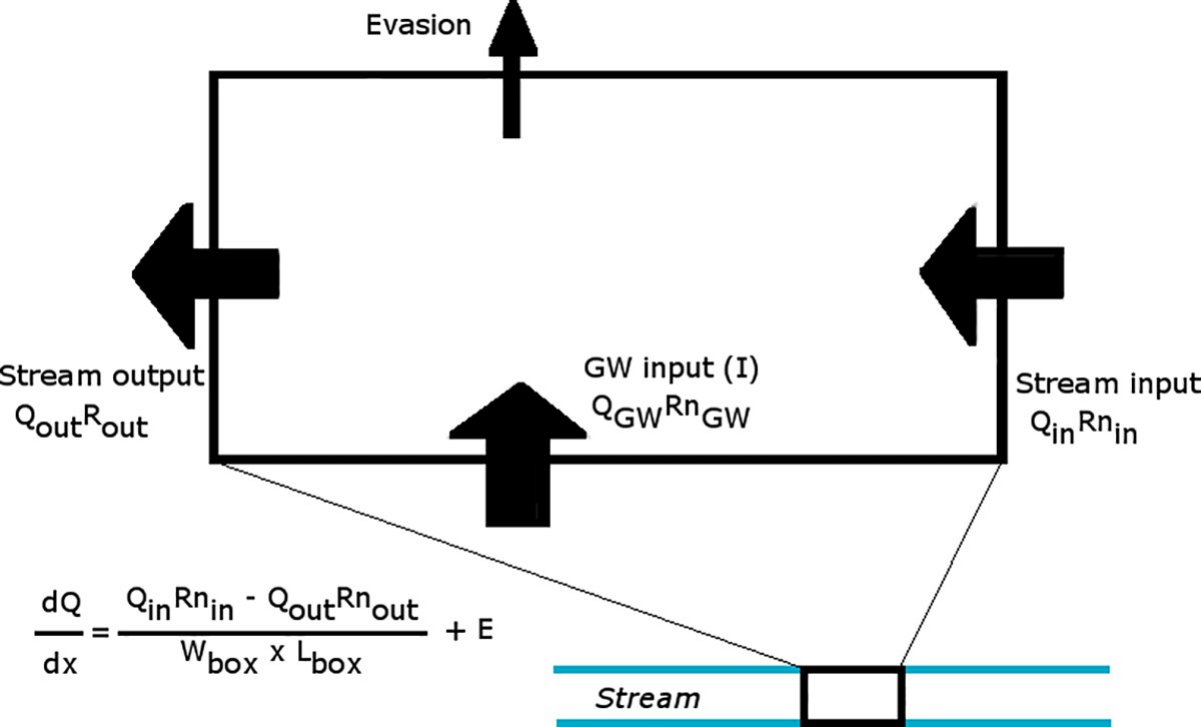
Radon box model used to calculate groundwater fluxes in streams. Groundwater discharge was calculated for each measured segment from upstream to downstream.

Groundwater fluxes in streams were calculated using a radon mass balance (Fig 3) in regular intervals (here called boxes) along the stream using Eq 4 (after [55], where $\frac{dQ}{dx}$ is the change in stream discharge per box, Q is the flux in/out measured during the seepage runs (m^3^ day^-1^), Rn is the radon concentration in/out (Bq m^-3^), w and L are width and length of the box (in m), and E accounts for evasion (Bq m^2^ day^-1^) and was calculated accounting for wind speed, current speed, and stream depth [52].


$$\frac{dQ}{dx}=\frac{Q_{in}{Rn}_{in}-Q_{out}{Rn}_{out}}{W_{box}L_{box}}+E$$ (4)

Radon survey data were corrected to account for the delay in radon air-water equilibration in the RAD-AQUA apparatus and ingrowth of its decay products (hereafter referred to as “modeled” radon). This was done by establishing the kinetic delay between radon in water and radon in air concentrations in laboratory experiments and applying those to correct for the kinetic and decay delay in field data [58]. For comparison and sensitivity analysis modeled results as well as “non-modeled” results, which did not apply the additional corrections for the kinetic and decay delay, were used to derive groundwater fluxes.

Groundwater discharge for radon time-series data were determined using a transient mass-balance model [53]. To calculate radon inventories, excess radon (in excess of ^226^Ra produced) activities were calculated. These inventories were then corrected for flood and ebb tides, mixing losses, and atmospheric evasion for each time step. Radon fluxes (Bq m^2^ day^-1^) were converted to groundwater fluxes (m^3^/day) by dividing the radon flux by the local maximum groundwater end-member radon concentration. Groundwater radon concentrations were measured for each segment of the coastline. Nutrient fluxes were calculated by multiplying discharge by nutrient concentrations measured in groundwater from beach porewater samples.

## Results

### Radon surveys and groundwater sampling

**Fig 4:**
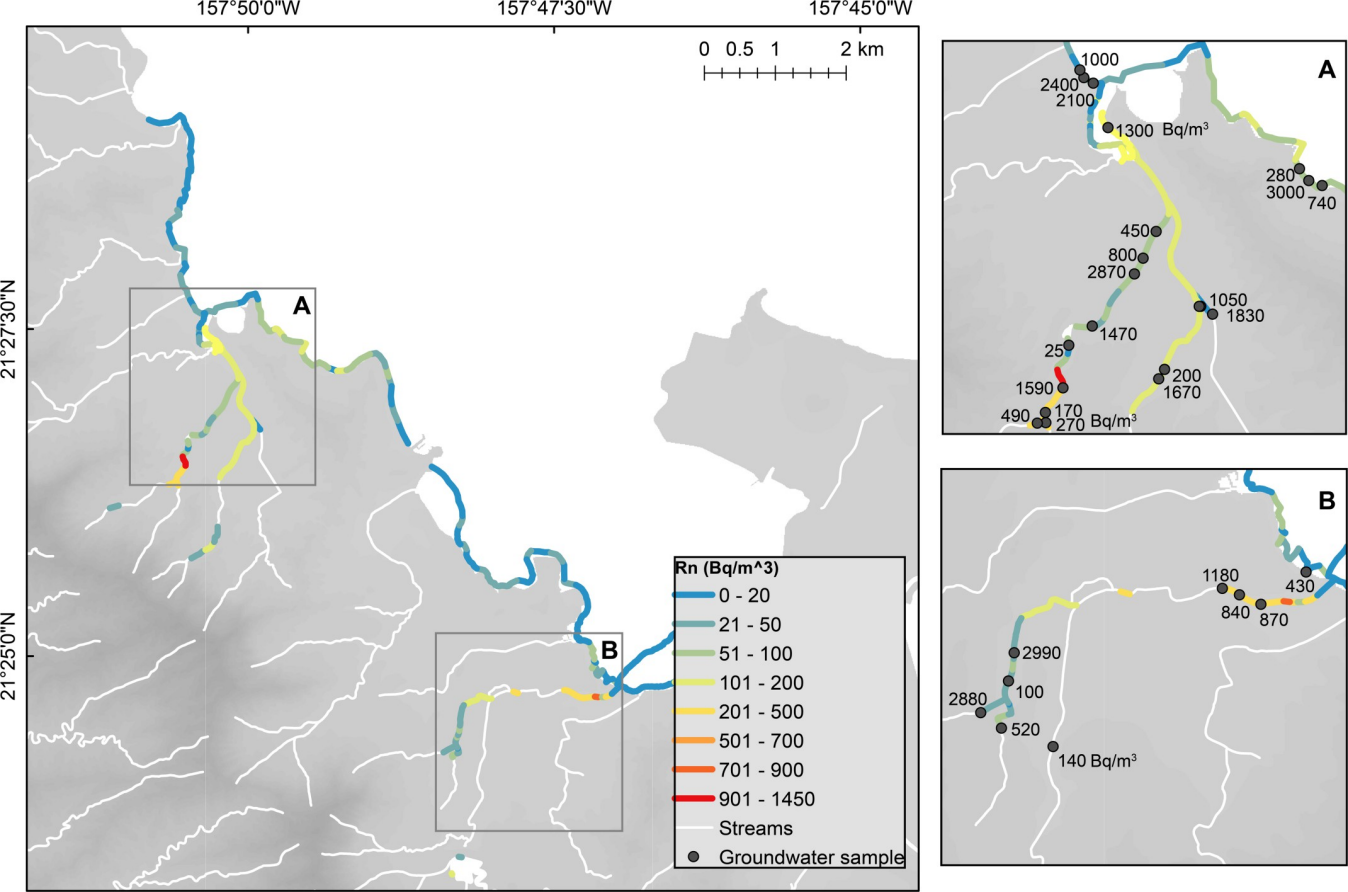
Non-modeled coastal and stream surface radon (Bq/m^3^) concentrations for Kāne’ohe Bay and studied streams (July sampling period). Stream surface radon concentrations (lines) and discrete (dots) groundwater radon concentrations are shown for (A) Kahalu’u and ’āhuimanu and (B) Kāne’ohe areas.

Coastal radon concentrations and SGD rates were spatially variable. Radon concentrations for all of Kāne’ohe Bay shoreline water ranged from 20 to 330 Bq/m^3^ (median = 88 Bq/m^3^) and 150 to 3,050 Bq/m^3^ (median = 980 Bq/m^3^) in coastal surface and beach face groundwater samples (Fig 4), respectively, and also varied by sector (S1 Table; S2 Table).

SGD fluxes using non-modeled radon concentrations ranged from 4,500 to 23,000 m^3^/day per sector (Table 5), and were greatest in the southern sector of the bay. In comparison, SGD fluxes estimated using modeled radon concentrations were only about three to four percent greater than non-modeled estimates so only the non-modeled will be considered in further discussion (S4 Table). Factoring in the shoreline length (in km) for each sector, SGD fluxes were lowest in the northwestern sector and greatest in the southern sector (Table 5).

**Table 5:** SGD fluxes.

SGD (10^4^ m^3^/day)				GW (mol/day)			
Sector	Samping Period	Q_SGD_	Q_Stream_	Q_DIN_	Q_DIP_	Q_DSi_	Q_DON_
		*SGD/km shoreline*		*Me*	*Me*	*Me*	*Me*
Northwest	July	0.45 ± 0.20	11	780 ± 1,300	47 ± 52	4,400 ± 2,100	360 ± 360
		*1*,*400*		*49*	*1*.*8*	*640*	*96*
Central	July	1.8 ± 1.1	0.63	690 ± 260	0.40 ± 0.30	1,700 ± 710	100 ± 35
		*3*,*900*		*240*	*0*.*27*	*500*	*310*
South	July	2.3 ± 1.9	2.3	670 ± 340	8.4 ± 9.5	5,500 ± 920	670 ± 1,500
		*4*,*000*		*130*	*1*.*6*	*780*	*92*

SGD and stream fluxes for the July sampling period, and associated DIN, DIP, DSi, and DON fluxes by sector. SGD (m^3^/km/day) per km of shoreline and median (Me) nutrient concentrations used for nutrient flux calculations are shown in italics. Modeled SGD fluxes (not shown; S4 Table) were within 4% of the non-modeled fluxes. Stream discharge data averaged by sampling period and location from USGS stream gage data [48]. The northwestern sector includes Waikāne, Waiāhole, Waihe’e, and Kahalu’u Streams, the central sector includes He’eia Stream, and the southern sector includes Kāne’ohe and Kawa Streams.

In streams, radon concentrations and groundwater discharge rates differed on both spatial and seasonal scales. For all studied sub-watersheds, radon concentrations in streams ranged from 21 to 3,400 (median = 270 Bq/m^3^) in surface, and from 23 to 3,500 (median = 940 Bq/m^3^) in groundwater samples. Median radon concentrations varied between sampling periods by sub-watershed (S3 Table). For Kahalu’u and ’āhuimanu sub-watersheds, radon concentrations in both surface and groundwater were lower during the February sampling period compared to the July sampling period. The opposite was true for Kāne’ohe sub-watershed. Groundwater fluxes in streams were calculated using both non-modeled and modeled results and a local radon end-member (S4 Table). Non-modeled groundwater fluxes ranged from 5,700 to 16,000 m^3^/day in the July sampling period and 6,600 to 17,000 m^3^/day in the February sampling period. Taking a conservative approach, we chose to use the non-modeled discharge rates for all subsequent calculations.

Baseflow (both in terms of volume and percentage of total stream flow) was greater during the February sampling period compared to the July sampling period for all three streams. Baseflow represented 49%, 22%, and 42% of total stream flow during the July sampling period and 68%, 40%, and 56% during the February sampling period for the studied sections of Kahalu’u, ’āhuimanu, and Kāne’ohe Streams, respectively. Baseflow during the July sampling period was well under the USGS estimate for baseflow (70% of total stream discharge). Of the streams studied, only baseflow during the February sampling period for Kahalu’u Stream was consistent with the USGS baseflow estimate.

### Nutrients in coastal and stream samples

Dissolved nutrient concentrations and fluxes varied by sector of Kāne’ohe Bay (Tables 5 and 6; S1 Table; salinity corrected concentrations in S5 Table). For coastal samples, dissolved nutrient concentrations in groundwater were statistically higher than corresponding concentrations in surface water for DIN, DON, DIP, and DSi according to the Kruskal-Wallis H-test. Coastal SGD nutrient fluxes were calculated as total SGD times the median nutrient concentrations in coastal groundwater, and were the greatest in the northwestern, and the least in the southern sectors of Kāne’ohe Bay for DIN and DIP, while DSi and DON fluxes were the greatest in the southern sector (Tables 5 and 6).

**Table 6:** Stream fluxes.

Streams (10^4^ m^3^/day)					GW (mol/day)				SW (mol/day)			
Stream	Sampling Period	Q_GW_	Q_SW_	Q_Stream_	Q_DIN_	Q_DIP_	Q_DSi_	Q_DON_	Q_DIN_	Q_DIP_	Q_DSi_	Q_DON_
		*% Q*_*Stream*_	*% Q*_*Stream*_		*Me*	*Me*	*Me*	*Me*	*Me*	*Me*	*Me*	*Me*
Kahalu’u	July	0.57 ± 0.28	0.66 ± 0.29	1.2	92 ± 79	4.4 ± 4.5	3,800 ± 1,600	260 ± 230	73 ± 51	5.5 ± 4.9	3,600 ± 320	36 ± 73
		*49%*	*51%*		*16*	*0*.*77*	*660*	*46*	*11*	*0*.*83*	*550*	*5*.*5*
	February	0.66 ± 0.19	0.32 ± 0.45	1.0	330 ± 380	3.8 ± 2.9	3,500 ± 1,900	290 ± 86	38 ± 4.8	2.7 ± 1.1	1,500 ± 240	17 ± 7.7
		*68%*	*32%*		*50*	*0*.*57*	*520*	*43*	*12*	*0*.*85*	*470*	*5*.*2*
’āhuimanu	July	0.67 ± 0.47	2.3 ± 0.47	2.9	110 ± 300	2.5 ± 3.9	4,500 ± 1,600	210 ± 150	160 ± 74	7.7 ± 6.4	11,000 ± 1,100	230 ± 110
		*22%*	*78%*		*16*	*0*.*37*	*670*	*31*	*6*.*9*	*0*.*34*	*490*	*10*
	February	0.95 ± 0.89	1.5 ± 0.88	2.4	110 ± 320	3.7 ± 3.1	5,700 ± 1,700	190 ± 93	110 ± 71	9.2 ± 7.7	6,900 ± 1,800	99 ± 56
		*40%*	*61%*		*12*	*0*.*39*	*600*	*20*	*7*.*5*	*0*.*61*	*460*	*6*.*6*
Kāne’ohe	July	1.6 ± 0.53	2.3 ± 0.84	3.9	420 ± 510	18 ± 21	8,200 ± 4,500	450 ± 1,100	390 ± 690	12 ± 8.1	12,000 ± 2,800	230 ± 370
		*42%*	*58%*		*26*	*1*.*1*	*510*	*28*	*17*	*0*.*54*	*520*	*10*
	February	1.7 ± 0.33	1.3 ± 0.80	3.1	540 ± 220	15 ± 7.1	7,800 ± 1,900	660 ± 880	160 ± 170	7.9 ± 3.3	6,200 ± 1,100	140 ± 79
		*56%*	*44%*		*31*	*0*.*83*	*450*	*38*	*12*	*0*.*61*	*480*	*11*

Groundwater (GW), surface water (SW), total stream fluxes and respective nutrient fluxes by sampling period and sub-watershed. Percentages in italics indicate the proportion that groundwater and surface water contribute to total stream discharge. Median nutrient concentrations used for flux calculations are indicated in italics underneath the respective nutrient flux.

In streams, dissolved nutrient concentrations were statistically higher in groundwater samples compared to surface samples for DIN, DON, and DSi, but were not statistically differentiable for DIP according to the Kruskal-Wallis H-test (Tables 5 and 6; S1 Table; S6 Table). Application of the same statistical test revealed that dissolved nutrient concentrations in stream and streambed-groundwater samples were not statistically differentiable between sampling periods. In-stream groundwater- and surface runoff-derived nutrient fluxes by season for the three studied sub-watersheds were highly spatially variable, particularly between groundwater and surface water fractions (Tables 5 and 6).

### Radon time series

**Fig 5:**
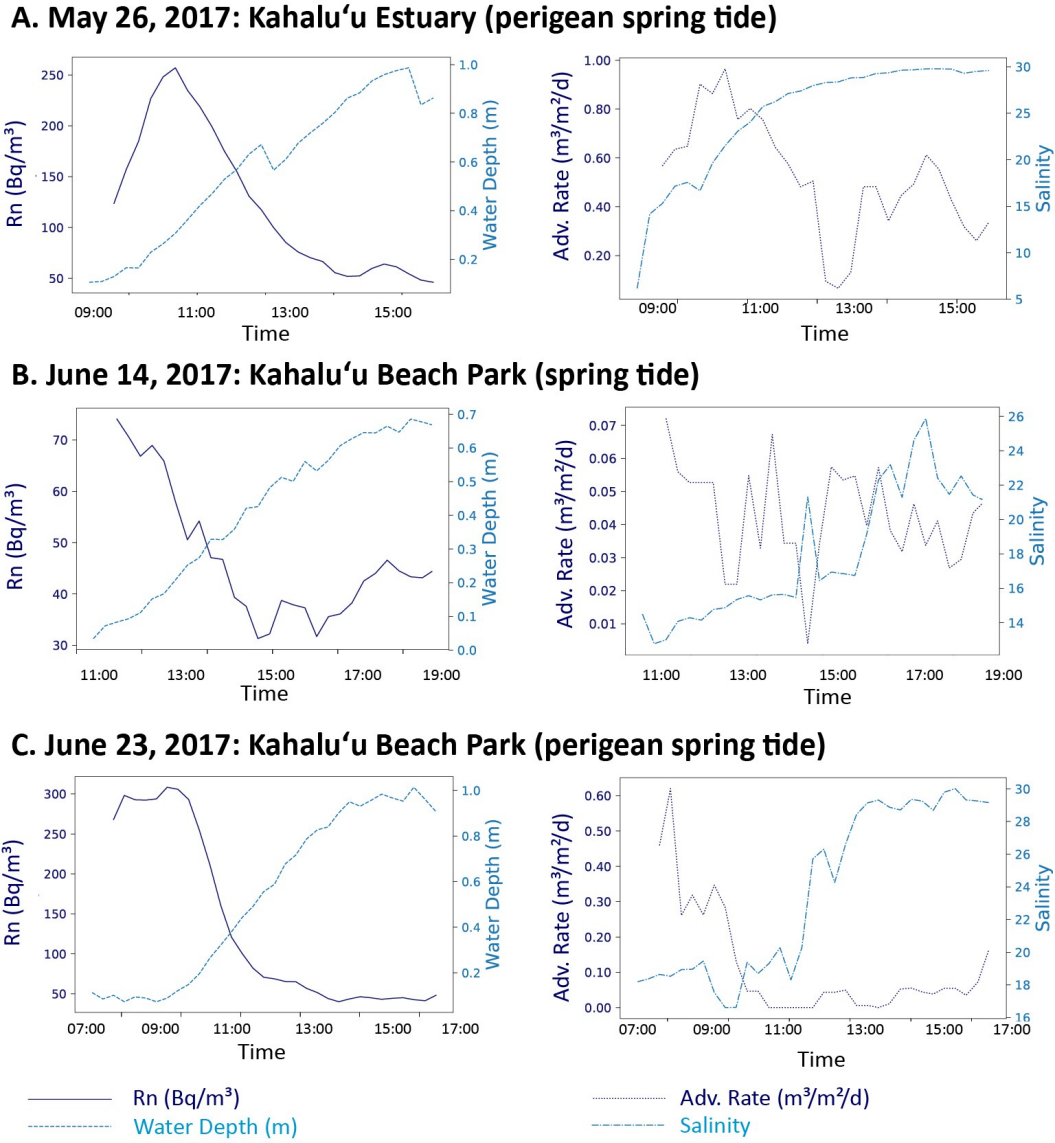
Radon time series. Radon (dark blue, in Bq/m^3^) and water depth (light blue, in m), and three-point running average advection rates (m^3^/m^2^/d) and salinity time series from low to high tide. Results from (A) May 26, 2017 (perigean spring tide, tidal range = 0.09 to 0.99 m) from Kahalu’u estuary, (B) June 14, 2017 (spring tide, tidal range = 0.03 to 0.69 m) from Kahalu’u Beach Park, (C) June 23, 2017 (perigean spring tide, tidal range = 0.02 to 1.01 m) from the same location at Kahalu’u Beach Park.

Three radon time series were conducted in Kahalu’u estuary over a half tidal cycle during May and June of 2017 (Fig 5). Salinities ranged from 6.0 to 30 (average = 25), 13 to 26 (average = 18), and 17 to 30 (average = 23) for the May 26, June 14, and June 23 sampling dates, respectively.

For the two time series conducted at Kahalu’u Beach Park, DIN, DSi, and DON concentrations and fluxes were greatest during low tide during the perigean spring tide (Table 7). Perigean spring tide nutrient fluxes averaged over the half tidal cycle were 3.6, 1.0, 1.7, and 6.9 times that of spring tide nutrient fluxes for DIN, DIP, DSi, and DON, respectively.

**Table 7:** Time series nutrient concentrations and fluxes sorted by tide.

		Concentration (μM)				(m^3^/d)	Flux (mol/d)			
		DIN	DIP	DSi	DON	SGD	DIN	DIP	DSi	DON
**ST**	LT	5.5	2.2	580	15	110	0.61	0.24	65	1.7
	HT	5.3	6.6	630	12	26	0.14	0.17	16	0.31
**KT**	LT	5.1	1.1	220	16	330	1.7	0.36	72	5.2
	HT	3.9	1.0	240	27	270	1.1	0.27	65	7.3
**KT:ST**	LT	0.92	0.48	0.39	1.1	3.0	2.7	1.5	1.1	3.1
	HT	0.73	0.16	0.38	2.4	10	7.7	1.6	4.0	23

Comparison of median dissolved nutrient (DIN, DIP, DSi, and DON) concentrations in groundwater, SGD, and nutrient fluxes between spring tide (ST) and perigean spring tide (KT) for samples collected at low (LT) and high (HT) tides at Kahalu’u Beach Park. Perigean spring tide to spring tide (KT:ST) concentrations and fluxes were greater during LT compared to HT. Nutrient concentrations were greater during the KT compared to the ST for DIN and DON. Nutrient fluxes were greater during the KT compared to the ST for DIN, DSi, DIP, and DON at both LT and HT.

For the two locations in Kahalu’u where radon time series were conducted, SGD rates were greatest at low tide. Anomalously high perigean tides resulted in greater total SGD fluxes at Kahalu’u Beach at both low and high tides compared to a typical summer spring tide at the same location (Table 8). June 14 (spring tide) coastal advection rates averaged at 0.04 ± 0.5 m^3^/m^2^/day with an average coastal salinity of 18 ± 3.8. June 23 (perigean spring tide) coastal advection rates averaged at 0.13 ± 0.24 m^3^/m^2^/day with an average coastal salinity of 23 ± 5.2. Advection rates were greater in Kahalu’u Estuary compared to the coastal ocean and average advection for the May 26 perigean spring tide was 0.54 ± 0.25 m^3^/m^2^/day with an average salinity of 24 ± 6.2. A substantially greater percentage of saline SGD was discharged during the perigean spring tide in comparison to the spring tide.

**Table 8:** Groundwater advection rates from radon time series.

Date	Tidal Range (m)	Low Tide Avg. Adv. Rate (m^3^/m^2^/d)	% Fresh GW	High Tide Avg. Adv. Rate (m^3^/m^2^/d)	% Fresh GW	Low Tide Avg. Salinity	High Tide Avg. Salinity
May 26, 2017	0.90–0.99*	0.54 ± 0.25	--	0.44 ± 0.18	--	15 ± 4.4	30 ± 0.2
June 14, 2017	0.03–0.69	0.06 ± 0.08	69%	0.04 ± 0.03	51%	14 ± 0.72	23 ± 1.8
June 23, 2017	0.02–1.01*	0.30 ± 0.36	47%	0.12 ± 0.12	14%	18 ± 1.0	29 ± 0.4

Average advection rates for low and high tides, percentage of fresh SGD, tidal range, and salinity for radon time series conducted at Kahalu’u Estuary and Beach.

* denotes a perigean spring tide.

## Discussion

### Review of the types and volumes of ground and surface water fluxes into Kāne’ohe Bay

#### Stream flow and the contribution of baseflow to total discharge

Streams are a significant source to Kāne’ohe Bay’s freshwater and nutrient budgets [27, 41]. For example, streams in the southern sector have been shown to supply 50% of the reactive nitrogen and almost all of the phosphate budget, albeit in form of particulate-bound organic compounds delivered during storm events [26, 41]. These authors also acknowledge that more studies should be focusing on groundwater as an additional nutrient pathway [41]. This study looked at streamflow in order to define what fraction of total stream discharge originates as baseflow from groundwater as well as determine the locations of these groundwater inflows within the watershed.

Total stream discharge was partitioned into baseflow and surface runoff. Because the aquifer structure in this watershed includes marginal dikes extending all the way to the shoreline (Fig 2), baseflow can be expected not only from the high-level and basal aquifers, but also between these zones through the dike structures. The marginal dike zone, although covered by alluvium, extends beneath the full length of the streambed. The baseflow reported here includes only that captured within the study region and mostly represents discharge from the basal lens through the alluvium. Disrepancies between baseflow estimates provided by the USGS and this study are likely the result of field work occurring during atypical climate conditions and by not capturing baseflow in the upper part of the watershed. Surface runoff or upstream baseflow not captured in this study comprised of about half of total stream flow for Kahalu’u and Kāne’ohe Streams, and represented nearly 80% of total stream flow for ’āhuimanu Stream during the dry season. During the February sampling period, the percentage of total stream flow represented by surface runoff decreased for all three streams.

**Fig 6:**
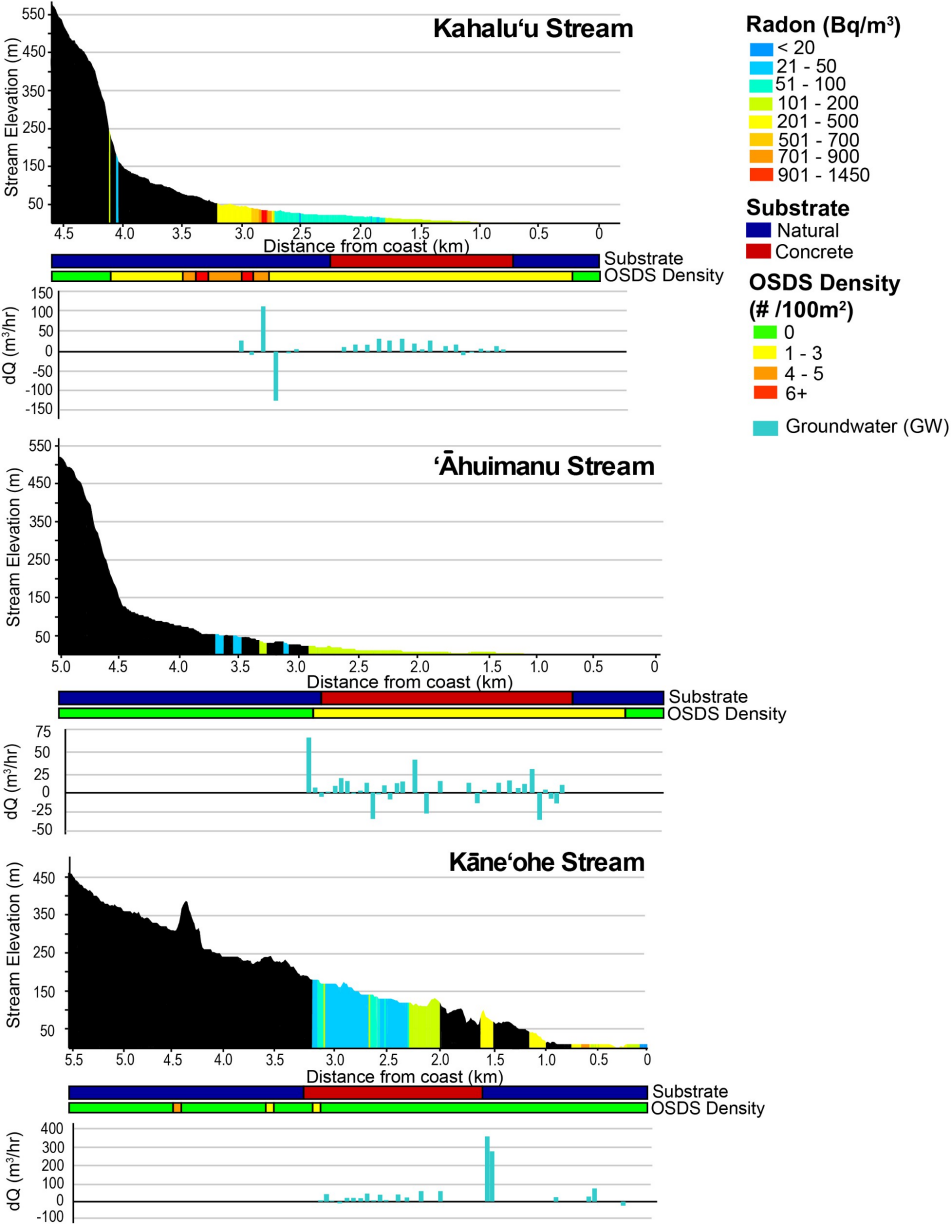
Groundwater fluxes in Kahalu’u, ’āhuimanu, and Kāne’ohe Streams (using non-modeled results) for the July sampling period. Stream elevation [59] is shown in m with surface water radon concentrations overlain in Bq/m^3^. Stream substrate (natural–blue, concrete–red) and the number of OSDS units within 100 m of the stream [32] are indicated below the graph showing stream elevation and radon concentrations. Corresponding changes in groundwater discharge are based on radon and stream discharge measurements.

The spatial distribution of groundwater inflows is heavily impacted by both geologic and anthropogenic factors. Substantial portions of the studied streams are lined with concrete, which alters surface water and groundwater flow paths and stream chemistry by disrupting hyporheic flow and decreasing water residence time resulting in faster flushing and less time for bioremediation [7]. Stream discharge, and particularly storm runoff, are accelerated in the concrete-lined portions due to the smooth, impervious surface. Geologically, for the streams studied, the highly conductive marginal dike zone intersects the streams upslope of most residential development, and is the major contribution of baseflow to the streams. Downstream of the marginal dike zone, older (low conductivity) and younger (low to moderate conductivity) alluvium prevails. The basal lens is the primary source of groundwater in these areas, however because a large portion of the streams are lined with concrete, groundwater inflows are inconsistent and limited to isolated locations (Fig 6). Groundwater contributions in these sections occur through drainage pipes, weepholes, and springs through cracks in the concrete-lining. Groundwater contribution in these outlets was confirmed based on their radon levels. In Kahalu’u Stream, residential areas with OSDS are concentrated within a 200 m radius of the stream, which also coincides with gaining portions of the stream (Fig 6). Losing reaches of Kahalu’u Stream are most significant in portions of the stream with a concrete substrate. Interestingly, this is not the case for ’āhuimanu Stream, where relatively high volumes of groundwater inflows and outflows occur within the concrete-lined section of the stream (Fig 6). This is likely the result of the numerous cracks observed within the concrete-lining, which were more pronounced within ’āhuimanu Stream compared to the others studied. Kāne’ohe Stream has mostly gaining reaches, particularly downstream of the portions lined with concrete (Fig 6).

Conclusions about groundwater fluxes in streams were inconsistent between using the modeled and non-modeled radon (S4 Table). Non-modeled and modeled groundwater discharge rates during the July and February sampling periods were within error of one another for Kahalu’u Stream. Similarly, non-modeled and modeled July results were comparable for Kāne’ohe Stream. July sampling period modeled (2.1 x 10^4^ m^3^/day) results for ’āhuimanu Stream however, were significantly greater than non-modeled (0.67 x 10^4^ m^3^/day) results. The discrepancy for ’āhuimanu Stream may be attributed to rapid fluctuations in radon concentrations, causing the modeled results (which are calculated in part using weighted averages and cubic splines) to overestimate the concentration.

#### SGD

Total nearshore SGD was 0.45 ± 0.20, 1.8 ± 1.1, and 2.3 ± 1.9 x 10^4^ m^3^/day in the northwestern, central, and southern sectors, respectively; however, total bay-wide SGD was smaller volumetrically than stream inputs (Tables 5 and 6). While total SGD was less than streamflow, it still represents a significant contribution to the overall water budget. Total SGD measured in this study was less than previous SGD estimates (1.1 to 9.4 x 10^5^ m^3^/day) for the northwestern and central sectors using radon and radium [16] because this study only captured nearshore SGD within 50 to 100 m of the shoreline. Another study in the area that used a MODFLOW model to estimate SGD (3.1 x 10^4^ m^3^/day) matched our estimates for SGD much more closely [60]. For the southern sector, SGD rates were 50% lower than total stream flow. The central sector had the greatest SGD rates across the bay, which were nearly three times greater than total stream flow. For the northwestern sector, SGD was 50% lower than total stream discharge.

Recirculated SGD was the primary component of total nearshore SGD bay-wide. The fresh component of total SGD bay-wide was 5.4 x 10^3^ m^3^/day, or 12% of total SGD. The volume of fresh SGD was highly variable by sector and represented 45%, 1.2%, and 20% of total SGD to the northwestern, central, and southern sectors, respectively.

For the sub-watersheds studied, SGD and baseflow were significant terrestrial water sources to the bay. For Kahalu’u sub-watershed, 0.21 x 10^4^ m^3^/day of SGD (55% of which is fresh SGD), and 0.57 x 10^4^ m^3^/day of baseflow discharge into Kāne’ohe Bay, which together contribute nearly 1.2 times that of surface runoff fraction of stream flow. For Kāne’ohe sub-watershed, 0.45 x 10^4^ m^3^/day (75% fresh SGD), and 1.6 x 10^4^ m^3^/day of baseflow discharge into the bay, contributing nearly equal parts of groundwater and surface runoff fraction of stream flow. Overall, 2.9 x 10^4^ m^3^/day of groundwater discharges to the bay from the Kahalu’u and Kāne’ohe sub-watersheds via baseflow and SGD, making groundwater an equal source of water to surface flow from streams to the bay in these areas.

Total SGD accounting for offshore SGD can be estimated if we assume that the same radon concentrations would be measured as far as 200 m offshore as was previously observed [16]. If we extend our radon mass balance volumes to 200 m offshore and water depth 1.4 m as previously observed [16], offshore SGD is estimated as 22,000, 31,000, and 31,000 m^3^/day for the northwestern, central, and southern sectors respectively. Our offshore SGD estimates for the northwestern and central sectors (the southern sector was not included in that study) are consistent with those in previous research [16, 60]. Our offshore SGD estimates are 490%, 170%, and 130% of our nearshore SGD estimates for the northwestern, central, and southern sectors, respectively, suggesting that SGD plumes and discharge points may extend offshore and SGD is potentially a substantial portion of the water budget, with water fluxes greater than stream inputs for both the central and southern sectors.

Our SGD estimates are somewhat lower in comparison to other studies using radon conducted globally in highly conductive substrates. Mean total SGD for Kāne’ohe Bay in this study was 2.5 m^3^/m/day (maximum = 29 m^3^/m/day). This is comparable to some studies conducted in other locations in Hawai’i, such as in Maui, where mean total SGD rates of 1.1 to 6.9 m^3^/m/day were found [61], but significantly lower than discharge rates reported by other authors in Kona (96 m^3^/m/day) [62]. Further comparisons between previous SGD studies conducted in Hawai’i have been detailed extensively in the literature [63]. Mean SGD, for instance, in Mauritus ranged from 5.2 to 56 m^3^/m/day [18, 64]. Similarly, mean SGD for Manila Bay, Phillippines was 12 m^3^/m/day [65] and 15 m^3^/m/day for Taiwan [66]. Differences between our SGD estimates and other studies may be attributed to local and regional differences in hydrogeological substrates (such as hydraulic conductivity or structure) and the fact that a significant portion of groundwater is channeled into stream as baseflow.

### Dissolved nutrient concentrations and fluxes

While major water quality problems associated with large point sources of pollution in Hawai’i, such as the sewage effluent outfall to Kāne’ohe Bay in the 1960’s to 1980’s [30], have been eradicated, non-point source pollution sourced from OSDS and agriculture are currently the largest contributors that still negatively impact coastal water quality [21–22, 29, 61]. These pollutant sources can negatively impact coral reefs by shifting their accretion-erosion balance or contributing to the proliferation of invasive algae [23, 30, 67]. In Kāne’ohe Bay, the impact of OSDS has never been studied in detail and at a bay-wide scale across population and hydrogeological gradients. As described earlier, both, streams and SGD are a pathway of terrestrial groundwater and therefore of land-derived sources of nutrients.

The Hawai’i Department of Health (HDOH) nutrient water quality standards for streams during the dry season were exceeded for 61% of TN and 33% of TP samples during the July sampling period [68]. For the February sampling period, 70% of TN and 5% exceeded the HDOH nutrient water quality standards for the wet season [68]. Nutrient concentrations within streams exceeded HDOH water quality standards for TN and TP [68]. Median concentrations during the July sampling period in surface waters of TN for all three streams studied were greater than the dry season HDOH limit of 13 μM, and less than the TP dry season HDOH limit of 0.97 μM [68]. Only Kāne’ohe Stream exceeded the median wet season concentrations for TN (HDOH limit = 18 μM) and none of the median values for streams exceeded the wet season TP limit of 1.6 μM. Based on the locations of groundwater discharge and associated nutrient fluxes, it is obvious that water quality in both, streams and the coastal ocean can be impaired by groundwater contributions. Specific to Kahalu’u Stream and downstream sections of ’āhuimanu Stream, the substantial number of cesspools within 100 m of the stream itself mean that groundwater contributions likely reflect a wastewater source (Fig 6). In particular, DIN and DIP concentrations and fluxes in surface water drastically increases where OSDS density exceeds 100 OSDS/km^2^, a metric which indicates a high risk for groundwater contamination [69]. According to the Mann-Whitney Rank Sum Test, concentrations of DIN (p = 0.002) and DIP (p = > 0.001) in surface water are significantly greater in the upstream portions with high OSDS density compared to downstream portions. In Kahalu’u Stream, upstream portions have an OSDS density that exceeds 100 OSDS/km^2^, where surface water nutrient concentrations (median TN = 19 ± 19 μM and TP = 1.1 ± 0.25 μM) exceed the HDOH limits of 13 and 0.97 μM for TN and TP, respectively. Nutrient concentrations are also enriched in groundwater despite low population density but high OSDS density, further suggesting that groundwater flows to streams in this area are impacted by wastewater. Nutrient concentrations were comparatively lower in the downstream portions of Kahalu’u and ’āhuimanu Streams (median TN = 18 ± 9.5 μM and TP = 0.30 ± 0.19 μM), while median TN concentrations still exceeded the HDOH limit. Similarly, in Kāne’ohe Stream, downstream portions (where the Kamo’oali’i and Kapunahala tributaries merge; also the area with the highest population density) had significantly higher nutrient concentrations (median TN = 58 ± 58 μM and TP = 1.1 ± 1.2 μM) compared to upstream portions (median TN = 12 ± 17 μM and TP = 0.53 ± 0.28 μM). In particular, median TN concentrations were significantly higher compared to those measured from Kahalu’u and ’āhuimanu Streams, which is consistent with previous literature indicating correlations of excess nitrogen with increasing population [5, 70] although this area does not have high density of OSDS. These results demonstrate that elevated stream nutrient concentrations can be traced back to groundwater nutrient levels and thus wastewater inputs because (1) radon analyses demonstrate groundwater connectivity to the streams and (2) locations with elevated stream nutrient concentrations had correspondingly high groundwater nutrient concentrations.

Median salinity corrected nutrient concentrations in SGD were higher than those measured in stream groundwater samples, which suggest that groundwater accumulates nutrients as it flows downstream in the watershed. Groundwater along the shoreline was brackish (salinity ranged from 3.2 to 28), meaning another potential contributor of nutrients (both organic and inorganic) to groundwater is seawater intrusion [71]. For example, previous research found elevated nutrient concentrations in salty porewater in He’eia, suggesting that remineralization associated with oxygenated saltwater cycling through sediments can be a source of inorganic nutrients [72]. The median concentration of DIN in coastal groundwater was nearly three times greater than that of stream groundwater. Similarly, DIP and DON in coastal groundwater were nearly twice and four times greater than median concentrations of groundwater from streams. Coastal surface waters had elevated nutrient concentrations and SGD is likely one of the important sources of these nutrients given the high median concentrations in SGD relative to stream inputs. One of the potential sources for the high nutrient concentrations along the coastline is the prevalence of coastal OSDS systems in the area, which may be compromised due to shallow groundwater levels [32]. Another potential explanation for the higher nutrient concentrations in SGD as opposed to stream groundwater inputs is that SGD is a result of converging groundwater flow paths at the coastline and many of these paths may be comparatively longer and thus are accumulating more nutrients.

**Fig 7:**
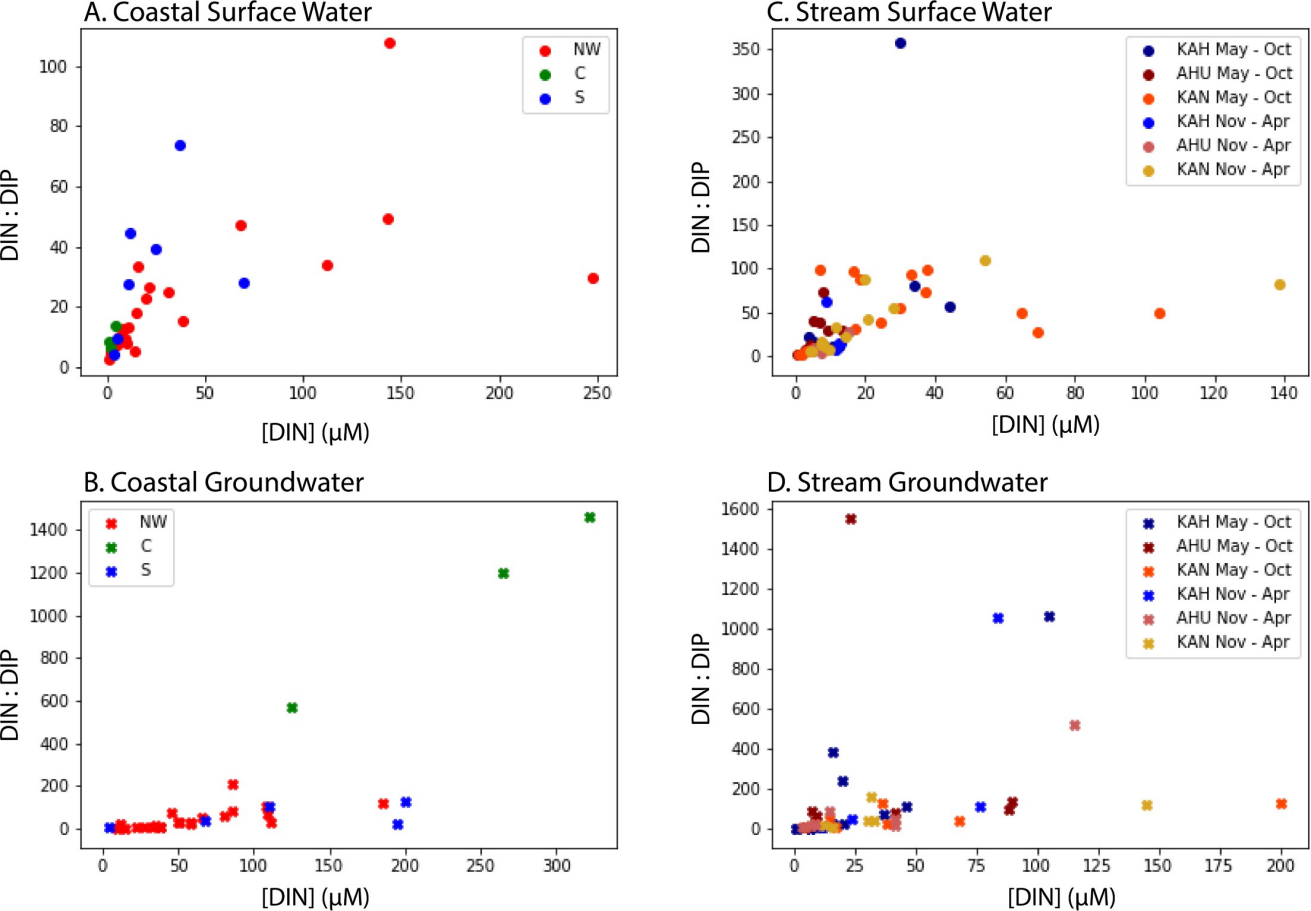
Salinity corrected DIN: DIP ratios vs. DIN concentrations by sector/sub-watershed, sampling period, and type of water. (A) Coastal surface water and (B) coastal groundwater, color-coded by NW, C, and S sectors. (C) Stream surface water and (D) stream groundwater, color-coded by sub-watershed: Kahalu’u (KAH), ’āhuimanu (AHU), and Kāne’ohe (KAN) and sampling period.

While median DIN and DIP concentrations did not vary between sampling periods in surface water, DIN: DIP ratios did vary between sampling periods in groundwater. DIN: DIP ratios can intensify from either an increase in nitrogen (e.g. sources from wastewater or fertilizers) or a decrease in phosphorus concentration or its increased sorption on aquifer solids. For Kahalu’u sub-watershed, the median DIN: DIP ratio in groundwater was over four times higher during the February sampling period (N:P = 83 ± 308), compared to the July sampling period (N:P = 21 ± 106; Fig 7), which given the potential sources of nitrogen in the sub-watershed, is likely a result of rainfall infiltrating the groundwater (and perhaps flooding the OSDS) and carrying excess nitrogen from the high density of OSDS in the sub-watershed [32]. The opposite trend was observed for ’āhuimanu sub-watershed, where the median DIN: DIP ratio in groundwater during the July sampling period (N:P = 82 ± 49) was greater than the February sampling period (N:P = 20 ± 45; Fig 7) due to a decrease in nitrogen concentrations. In Kāne’ohe sub-watershed, DIN: DIP ratios were similar between July (N:P = 24 ± 48) and February (N:P = 39 ± 75) sampling periods.

In the nearshore environment, median salinity corrected DIN: DIP ratios were variable by sector in surface and groundwater across the bay (Fig 7). The central sector had a median DIN: DIP ratio in surface waters less than the anticipated Redfield ratio of 16 [19] and thus N-limiting, consistent with previous research in the area [30, 73]. Despite a large number of OSDS units upstream, He’eia Stream flows into a wetland prior to discharging to the coastal ocean in the central sector and may be a significant removal term of nitrogen through denitrification as observed in other wetlands in similar environments [16]. In contrast to the central sector, surface waters in the northwestern and southern sectors had DIN: DIP ratios greater than Redfield ratios. For all of nearshore Kāne’ohe Bay, DIN: DIP ratios in groundwater were consistent with ranges found for SGD in other locations around the world, with a DIN: DIP ratio several orders of magnitude greater than the Redfield ratio [74].

Dissolved silica is primarily delivered to streams and the nearshore area via groundwater and is an essential nutrient for silica-based organisms. Silicates are sourced in Hawai’i almost exclusively from weathered basalt and soils [75]. Surface waters in streams naturally had higher median salinity corrected concentrations of DSi (median concentrations were 530, 480, and 500 μM for Kahalu’u, ’āhuimanu, and Kāne’ohe Streams, respectively) compared to coastal waters (median DSi concentrations were 440, 230, and 370 μM for the northwestern, central, and southern sectors, respectively) due to the contribution of baseflow to streams. Groundwater concentrations of salinity corrected DSi were greater in coastal groundwater (median concentrations were 690 and 860 μM for the north-western and southern sectors) compared to groundwater samples from streams (median concentrations were 600, 620, and 450 μM for Kahalu’u, ’āhuimanu, and Kāne’ohe Streams, respectively). Overall, stream surface water DSi concentrations found in this study are consistent with previous research in the area that stated that streams under baseflow conditions had 400 to 500 μM DSi on average [26].

**Fig 8:**
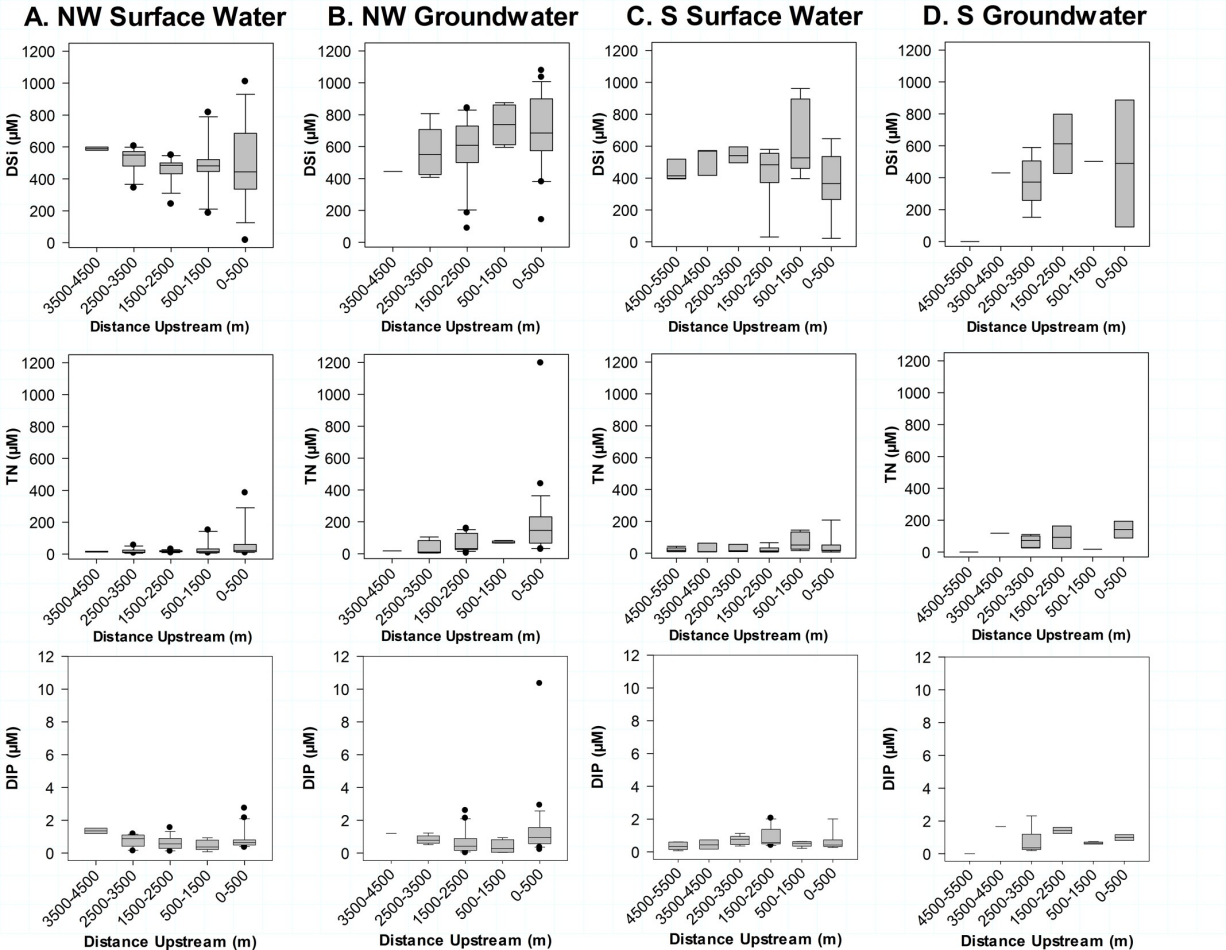
Salinity-corrected DSi, TN, and DIP (μM) boxplots by distance downstream (m) for the Kahalu’u and Kāne’ohe Stream-Coastal continuums. (A) Surface water and (B) groundwater for Kahalu’u Stream flowing into the NW sector. (C) Surface water and (D) groundwater for Kāne’ohe Stream flowing into the S Sector.

Along the stream-coastal continuum, nutrient additions show relationships between groundwater flow paths and localized inputs. The Kahalu’u Stream-coastal continuum shows that the streams are progressively gaining from the upstream reaches above the concrete-lined portion and then once again where Kahalu’u Stream flows into Kahalu’u Estuary to the coastal ocean, as evidenced with DSi concentrations (Fig 8). Coastal groundwater is enriched in DSi, which is consistent with the assumed deeper and longer groundwater flow path also surmised for increased nitrogen levels, DSi largely depends on groundwater residence time [76]. The lowest concentrations of DSi in groundwater samples within the concrete-lined portions of the stream (particularly at 1500–2500 m downstream) likely reflect input from storm drains, which is low in DSi. Additions of TN also increase with distance downstream, likely due to increased anthropogenic influence, such as OSDS density, in addition to longer groundwater flow paths forced by alteration of the stream’s natural substrate and channel (Fig 8). Concentrations of DIP in general show a decreasing trend with respect to distance downstream in both surface water and groundwater for the northwest sector, likely reflecting local inputs from OSDS as trends between (Fig 8). For the southern sector, DIP concentrations actually increase in surface waters with respect to distance downstream, until reaching the estuary, which instead is likely sourced from the decay of plants growing within the concrete lining of the streambed (Fig 8). The Kāne’ohe Stream-coastal continuum shows a similar trend (Fig 8), however it is complicated by several factors including higher population density, fewer springs within the concrete-lined section, and vegetation within the concrete-lined portions of the streams. Moreover, the highest concentrations of both TN and DIP coincide with downstream areas with the highest population density. The salinity correction surmises the assumption that a large fraction of the nutrient concentrations observed at the coast is terrestrially-derived, but may over-estimate the concentration if the source of the input is at the shoreline itself to brackish waters (such as OSDS units located on the coastline).

Salinity-corrected nutrient fluxes to the northwestern and southern sectors of Kāne’ohe Bay were primarily groundwater (SGD and stream baseflow) derived (Table 9). For both sectors studied, DIN and DON fluxes were primarily delivered to the bay via groundwater (SGD and baseflow combined). Previous research indicated that 3, 1, 0.1, and 26 kmol/day of DIN, DON, DIP, and DSi, respectively are loaded to the southern sector of Kāne’ohe Bay [30]. Our results for southern Kāne’ohe Bay closely match these fluxes (Table 9), with the exception of DSi, which is 46% (12 kmol/day) greater. This discrepancy may be due to the increased development and erosion or previous underestimation of SGD fluxes.

**Table 9:** Total (SGD and stream) dissolved nutrient loading to Northwestern (NW) and Southern (S) Kāne’ohe Bay during the dry season.

		Total (kmol/d)	% SGD	% Stream	% Groundwater
NW	DIN	0.38	58	42	81
	DON	0.73	59	41	95
	DIP	0.018	45	55	69
	DSi	10	28	72	65
S	DIN	3.8	79	21	90
	DON	2.8	76	24	92
	DIP	0.067	55	45	82
	DSi	38	47	53	69

% SGD and % Stream represent the percentage contribution to the total groundwater-derived nutrient flux for DIN, DON, DIP, and DSi for the high resolution study areas. % Groundwater refers to the percentage that SGD and stream baseflow contribute to the total (groundwater + surface water) nutrient flux.

### Temporal variation of SGD during normal and extreme tidal cycles

The above reported bay-wide SGD rates represent only a snapshot of discharge rates. It is well documented that SGD variation is driven by tides. For the two locations in Kahalu’u where radon time series were conducted, SGD rates were greatest at low tide, in accordance with previous SGD studies in Hawai’i [16, 22, 61]. A substantially greater percentage of saline SGD was discharged during the perigean spring tide in comparison to the spring tide, which is consistent with previous literature suggesting increased saline SGD at high sea level stands [4]. These temporal variations also have implications for SGD flux estimates, where our results from Kahalu’u suggest that bay-wide SGD would likely be significantly greater during a perigean spring tide compared to a spring tide and especially compared to a neap tide.

Anomalously high tides offer a snapshot into future coastal scenarios given projected sea levels in the next 30 to 100 years [77–78], where potential examples of impacts include coastal nuisance flooding and inundation of OSDS [79]. The higher rates of groundwater discharge and nutrient fluxes observed during the perigean spring tide suggest that these impacts are highly likely to be occurring today during high tide cycles, which has important implications for coastal biogeochemical systems. Non-point pollution sources, such as OSDS, in Kāne’ohe Bay and within the state of Hawai’i, are frequently located along coastlines, meaning coastal water quality will likely worsen with increasing sea-levels due to the inundation of these systems.

Increasing population and development along coastlines coupled with projections of increased global mean sea level (conservatively, 0.3 to 1 m within the next 100 years) may exacerbate future coastal water quality deterioration, not only in Hawai’i and on HVPI, but also globally [80–81]. This study is one of the first, to our knowledge, to directly study the impact of increasing sea levels on SGD discharge rates and associated nutrient fluxes. We observed an increase in total SGD, which has important implications for coastal ecosystems. Increases in total SGD allow for higher rates of groundwater discharge, and contaminants associated with land use that travel via groundwater, such as excess nutrients and sewage, to reach the coastal zone. While we observed a higher percentage of saline SGD during the perigean spring tide, low tide fresh SGD was still 3.4 times greater during the perigean spring tide compared to the spring tide (Table 8). Moreover, increased saline SGD may promote dissolution of metals and dissolved species, representing a potential additional source of contamination to the coastal ocean [2].

In this study, we have shown not only increased nutrient fluxes, but also higher nutrient concentrations during perigean spring tides, highlighting the importance of conducting more studies that investigate the relation of sea level to SGD composition. In particular, an excess in nitrogen sources were observed during the perigean spring tide compared to the spring tide, dramatically increasing the N:P ratio from 7.0 to 58 during low tide and from 1.4 to 26 during high tide (Table 7). Given these results, we suggest that rising sea levels may disrupt primary productivity with greater frequency due to increasing departure from the Redfield ratio.

### Groundwater-surface water interactions along the stream-coastal continuum

This study takes a novel approach by looking at groundwater baseflow and SGD as a continuous vector for pollution via groundwater flow. Groundwater contributions to streams ranged from 22% to 68% along their studied reaches to the coast of Kāne’ohe Bay and Watershed, whereas nearshore SGD ranged from 1,400 to 4,000 m^3^/km/day, or 9% to 58% of groundwater discharged to the studied streams as baseflow. This is not surprising as streams intercept the aquifer and gain a significant amount of groundwater in the watershed, draining groundwater from the aquifers. In a ridge to reef concept, our results suggest that groundwater discharge is important for both the water and nutrient budgets of the studied reaches of the streams, estuaries, and coastal ocean.

Both streams and the coastal SGD are important vectors for nutrient delivery to Kāne’ohe Bay. Groundwater contributions of DIN, DIP, and DSi in streams discharging to Kāne’ohe Bay were 23%, 58%, and 46% of total stream inputs during the dry season, respectively. For the sub-watersheds in which stream inputs were measured, SGD contributes 810, 15, and 6,400 moles/day, or 83%, 38%, and 23% of DIN, DIP, and DSi, respectively compared to stream fluxes. This illustrates the importance of considering both baseflow and SGD as vectors of groundwater pollution to the coastal ocean. Groundwater-derived DON contributions were 85% of total stream flow, whereas SGD-derived DON added 840 moles/day, or 86% of stream inputs.

Salinity corrected coastal groundwater concentrations were mostly similar or greater compared to results from previous SGD studies conducted in Hawai’i. Our median bay-wide coastal groundwater DIN concentrations (62 μM) were greater than mean values measured in other SGD studies in Kona, Southern Moloka’i, and Kāne’ohe Bay, similar to those measured in Wailupe but less than concentrations measured at Black Point and on Maui (Table 10), likely due to the suspected wastewater influence from OSDS, but comparatively less than sites such as Black Point and West Maui. Similarly, our coastal groundwater DIP concentrations (1.6 μM) were greater than those measured in Kona and Moloka’i, Wailupe, and Kāne’ohe Bay, and less than those measured in Black Point, and on West Maui that have known OSDS pollution (Table 10). Median bay-wide DSi concentrations (640 μM) in coastal groundwater were greater than average concentrations found on Maui, Kona, Moloka’i, but similar to those measured previously in Kāne’ohe Bay, and less than the average concentrations from Black Point and Wailupe (Table 10), which are likely associated with SGD rates (for Black Point and Wailupe) and island age and weathering (for sites on Maui, Moloka’i, and Hawai’i).

**Table 10:** Comparison of coastal groundwater nutrient concentrations between this study and other studies in Hawai’i.

	DIN (μM)	DIP (μM)	DSi (μM)	References
This Study	62	1.6	640	
Kāne’ohe Bay, O’ahu	12	1.6	540	[16]
Wailupe, O’ahu	71	1.7	810	[22]
Black Point, O’ahu	160	3.7	740	[22]
Kona, Hawai’i	14–39	0.83–1.8	110–210	[82] [83]
West Maui	120	3.0	510	[59]
Kamiloloa, Moloka’i	3.9	0.89	47	[83]

Salinity corrected coastal groundwater nutrient concentrations in the nearshore waters were comparable to or greater than the global fresh SGD concentrations. The median DSi concentration in coastal waters was about five times greater compared to the global fresh SGD DSi end-member value of 130 ± 18 μM [74]. Similarly, the median bay-wide DIP concentration was over twice the global end-member value for DIP (0.6 ± 0.2 μM) [74]. This is not surprising as DSi and DIP are reported to be elevated in basalt aquifers [84, 85, 86]. The median coastal DIN concentration found in this study however, was consistent with the global DIN end-member concentration (56 ± 23 μM) [74].

The results from this study have important implications for our understanding of groundwater discharge to coastal environments, especially in areas subject to stream discharge. This is particularly the case for volcanic or karstic settings, which while dissimilar geologically, have similar hydrogeologic properties such as high permeability and porosity that lead to enhanced groundwater discharge [18, 87]. Channelization of streams coupled with a hydrologically conductive substrate resulted in increased groundwater discharge and nutrient fluxes, particularly to the coastal ocean. Parsing total groundwater and surface water contributions leads to more informed land management decisions, which will become increasingly important in coming years under higher sea level stands.

## Conclusion

Partitioning groundwater and surface water discharge along the stream-coastal continuum allowed for a greater spatial and temporal resolution of groundwater discharge dynamics, particularly in areas with substantial baseflow contribution to streams. Most studies have largely focused solely on either baseflow to streams and SGD to the coastal ocean as separate entities. While fresh SGD represents an estimated 10% of river discharge globally [11], we suggest baseflow contributions to streams represent an important, yet understudied, addition to coastal groundwater budgets. The approach used in this study not only led to an improved understanding of nutrient delivery and contaminant flow paths to streams and the coastal ocean in Kāne’ohe Watershed and Bay, but also highlighted the importance of considering groundwater discharge via stream baseflow. Our major findings include:

1. Groundwater (stream baseflow + SGD) fluxes were equal to surface runoff for the studied streams, which demonstrates the importance of considering groundwater contributions to both, streams and the coastal ocean in water and geochemical budgets.
2. SGD-derived nutrient concentrations and fluxes were greater than stream-derived nutrient fluxes. In particular, nitrogen species were high in SGD, shifting nearshore N:P ratios substantially higher than conditions that promote balanced primary productivity.
3. SGD fluxes during a perigean spring tide were greater than those of a spring tide at the same location. Similarly, DIN, DON, DIP, and DSi fluxes were greater during the perigean spring tide. Sea level rise will stress coastal infrastructure globally—attempting to understand these impacts through field-based studies will help prepare individuals, land-managers, and governments, in addition to improving available data for modelers, for the future.

This research highlights the importance of considering groundwater discharge as a continuous water and solute source across the land-ocean interface, in addition to being one of the first field-based studies to look at groundwater discharge dynamics and contaminant transport under future sea level stands. We recommend future SGD studies in areas that are influenced by stream discharge use a similar approach to the one used in this study in order to account for total groundwater discharge to the coastal ocean, particularly in volcanic and karstic substrates. As demonstrated by this study, water quality in streams and the coastal ocean are linked by groundwater discharge. While it is often difficult to detect coastal springs without specific SGD detection methods that are not available to all monitoring agencies, this work suggests that because of the similarities between groundwater discharge-driven coastal and estuarine water quality, estuarine monitoring often captures the groundwater signature and may inform about sources of coastal water quality problems as well.

## Supporting information

S1 Table Raw data for all grab samples and radon survey samples in this study. Grab sample data are categorized by type (groundwater–gw, surface water–surf, and well) and location. Date collected and lat long indicate the sampling date and location of sampling. Rn concentrations (Bq/m^3^), temperature (°C), and salinity are given for each grab sample. Additionally, nutrient data (TN, TP, DIP, DSi, NOx, NH_4_^+^, DIN, and DON) are provided in μM. Radon survey data show location (lat long), date and time of measurement, water depth (m), salinity, and temperature (°C), and the radon concentration (Bq/m^3^).
(XLSX)

S2 Table Median radon concentrations in coastal grab samples. Radon concentrations are in Bq/m^3^ ± the interquartile range (IQR) by sector of Kāne’ohe Bay.
(DOCX)

S3 Table Median radon concentrations in stream grab samples. Radon concentrations are in Bq/m^3^ ± IQR for the July and February sampling periods, by sub-watershed.
(DOCX)

S4 Table Modeled vs. non-modeled groundwater fluxes. **(A)** Modeled SGD fluxes in 10^4^ m^3^/day for the July sampling period, percentage difference between SGD fluxes using non-modeled and modeled radon by sector of Kāne’ohe Bay. **(B)** Modeled groundwater (GW) fluxes in 10^4^ m^3^/day, by sampling period and sub-watershed. Percentages indicate the proportion that groundwater and surface water contribute to total stream discharge.
(DOCX)

S5 Table Salinity-corrected nutrient concentrations for coastal samples. Median concentrations (μM) ± IQR of salinity-corrected nutrients for coastal samples by Kāne’ohe Bay sector and water type.
(DOCX)

S6 Table Salinity-corrected nutrient concentrations for stream samples. Median concentrations (μM) ± IQR of salinity-corrected nutrients by sub-watershed and water type for samples collected during dry and wet seasons.
(DOCX)
