# Supplementary material for: Parallels between stream and coastal water quality associated with groundwater discharge
Source: PLoS One. 2019 Oct 28;14(10):e0224513. doi: 10.1371/journal.pone.0224513 (PMC6816572; doi:10.1371/journal.pone.0224513)
Supplement: S2 Table — Radon concentrations are in Bq/m3 ± the interquartile range (IQR) by sector of Kāne’ohe Bay. (DOCX) [file pone.0224513.s002.docx]

**S1 Table. Median radon concentrations in coastal grab samples.**

| **Location** |  | **Median Rn** |
| --- | --- | --- |
| Northwest | Ground  (n = 25) | 1,400 ± 1,200 |
|  | Surface  (n = 23) | 95 ± 150 |
| Central | Ground  (n = 3) | 390 ± 270 |
|  | Surface  (n = 4) | 43 ± 17 |
| South | Ground  (n = 5) | 860 ± 590 |
|  | Surface  (n = 7) | 170 ± 220 |

Radon concentrations are in Bq/m^3^ ± the interquartile range (IQR) by sector of Kāneʻohe Bay.
