# Supplementary material for: Parallels between stream and coastal water quality associated with groundwater discharge
Source: PLoS One. 2019 Oct 28;14(10):e0224513. doi: 10.1371/journal.pone.0224513 (PMC6816572; doi:10.1371/journal.pone.0224513)
Supplement: S3 Table — Radon concentrations are in Bq/m3 ± IQR for the July and February sampling periods, by sub-watershed. (DOCX) [file pone.0224513.s003.docx]

**S2 Table.** **Median radon concentrations in stream grab samples.**

| **Location** |  | **July Sampling Period Rn** | **February Sampling Period Rn** |
| --- | --- | --- | --- |
| Kahaluʻu | ground | 800 ± 1,020  (n = 13) | 730 ± 860  (n = 4) |
|  | surface | 310 ± 160  (n = 12) | 85 ± 290  (n = 4) |
| ʻĀhuimanu | ground | 1,050 ± 1,200  (n = 8) | 480 ± 340  (n = 8) |
|  | surface | 590 ± 690  (n = 11) | 130 ± 330  (n = 3) |
| Kāneʻohe | ground | 900 ± 1,200  (n = 8) | 1,900 ± 1,200  (n = 6) |
|  | surface | 140 ± 250  (n = 19) | 250 ± 230  (n = 13) |

Radon concentrations are in Bq/m^3^ ± IQR for the July and February sampling periods, by sub-watershed.
