# Supplementary material for: Parallels between stream and coastal water quality associated with groundwater discharge
Source: PLoS One. 2019 Oct 28;14(10):e0224513. doi: 10.1371/journal.pone.0224513 (PMC6816572; doi:10.1371/journal.pone.0224513)
Supplement: S4 Table — (A) Modeled SGD fluxes in 104 m3/day for the July sampling period, percentage difference between SGD fluxes using non-modeled and modeled radon by sector of Kāne’ohe Bay. (B) Modeled groundwater (GW) fluxes in 104 m3/day, by sampling period and sub-watershed. Percentages indicate the proportion that groundwater and surface water contribute to total stream discharge. (DOCX) [file pone.0224513.s004.docx]

**S3 Table. Modeled vs. non-modeled groundwater fluxes.**

| **A.** | **Sector** | **Sampling Period** | **SGD (modeled)** | **Non-modeled vs. modeled Rn (%)** |
| --- | --- | --- | --- | --- |
|  | Northwest | July | 0.46 | 97 % |
|  | Central | July | 1.9 | 97 % |
|  | South | July | 2.3 | 96 % |

| **B.** | **Stream** | **Sampling Period** | **GW (modeled)** | **Non-modeled vs. modeled Rn (%)** |
| --- | --- | --- | --- | --- |
|  | Kahaluʻu | July | 0.54 | 106 % |
|  |  | February | 0.52 | 127 % |
|  | ʻĀhuimanu | July | 2.1 | 31.9 % |
|  |  | February | 0.65 | 146 % |
|  | Kāneʻohe | July | 1.6 | 100 % |
|  |  | February | N/A | N/A |

(A) Modeled SGD fluxes in 10^4^ m^3^/day for the July sampling period, percentage difference between SGD fluxes using non-modeled and modeled radon by sector of Kāneʻohe Bay. (B) Modeled groundwater (GW) fluxes in 10^4^ m^3^/day, by sampling period and sub-watershed. Percentages indicate the proportion that groundwater and surface water contribute to total stream discharge.
