# Supplementary material for: Parallels between stream and coastal water quality associated with groundwater discharge
Source: PLoS One. 2019 Oct 28;14(10):e0224513. doi: 10.1371/journal.pone.0224513 (PMC6816572; doi:10.1371/journal.pone.0224513)
Supplement: S5 Table — Median concentrations (μM) ± IQR of salinity-corrected nutrients for coastal samples by Kāne’ohe Bay sector and water type. (DOCX) [file pone.0224513.s005.docx]

**S4 Table. Salinity-corrected nutrient concentrations for coastal samples.**

| **Location** |  | **DIN** | **DIP** | **DSi** | **DON** |
| --- | --- | --- | --- | --- | --- |
| Northwest | ground  (n = 24 ) | 50 ± 54 | 1.8 ± 2.2 | 690 ± 300 | 93 ± 100 |
|  | Surface  (n = 23) | 14 ± 6.9 | 0.84 ± 0.72 | 440 ± 230 | 8.1 ± 9.7 |
| Central | ground  (n = 3) | 260 ± 98 | 0.27 ± 0.20 | 530 ± 230 | 330 ± 120 |
|  | surface  (n = 4) | 3.4 ± 28 | 0.24 ± 0.22 | 230 ± 77 | 11 ± 37 |
| South | ground  (n = 5) | 110 ± 130 | 1.6 ± 0.85 | 850 ± 120 | 7.0 ± 190 |
|  | surface  (n = 5) | 12 ± 23 | 0.59 ± 0.27 | 370 ± 220 | 7.5 ± 4.6 |

Median concentrations (µM) ± IQR of salinity-corrected nutrients for coastal samples by Kāneʻohe Bay sector and water type.
