# Supplementary material for: Parallels between stream and coastal water quality associated with groundwater discharge
Source: PLoS One. 2019 Oct 28;14(10):e0224513. doi: 10.1371/journal.pone.0224513 (PMC6816572; doi:10.1371/journal.pone.0224513)
Supplement: S6 Table — Median concentrations (μM) ± IQR of salinity-corrected nutrients by sub-watershed and water type for samples collected during dry and wet seasons. (DOCX) [file pone.0224513.s006.docx]

**S6 Table.** **Salinity-corrected nutrient concentrations for stream samples.**

| **Location** |  | **DIN** | **DIP** | **DSi** | **DON** |
| --- | --- | --- | --- | --- | --- |
| **July Sampling Period** | | | | | |
| Kahaluʻu | ground (n = 13 ) | 16 ± 14 | 0.77 ± 0.80 | 660 ± 280 | 46 ± 41 |
|  | surface (n = 12 ) | 11 ± 7.8 | 0.83 ± 0.74 | 550 ± 48 | 5.5 ± 11 |
| ʻĀhuimanu | ground (n = 8) | 16 ± 45 | 0.37 ± 0.58 | 670 ± 240 | 31 ± 22 |
|  | surface (n = 11) | 6.9 ± 3.2 | 0.34 ± 0.28 | 490 ± 49 | 10 ± 4.6 |
| Kāneʻohe | ground (n = 8) | 26 ± 32 | 1.1 ± 1.3 | 510 ± 280 | 28 ± 66 |
|  | surface (n = 19) | 17 ± 30.0 | 0.54 ± 0.35 | 520 ± 120 | 10.0 ± 16 |
| **February Sampling Period** | | | | | |
| Kahaluʻu | ground (n = 4) | 50 ± 57 | 0.57 ± 0.43 | 520 ± 280 | 43 ± 13 |
|  | surface (n = 4) | 12 ± 1.5 | 0.85 ± 0.34 | 470 ± 74 | 5.2 ± 2.4 |
| ʻĀhuimanu | ground (n = 8) | 12 ± 33 | 0.39 ± 0.33 | 600.0 ± 180 | 20.0 ± 9.8 |
|  | surface (n = 3) | 7.5 ± 4.7 | 0.61 ± 0.51 | 460 ± 120 | 6.6 ± 3.7 |
| Kāneʻohe | ground (n = 6) | 31 ± 13 | 0.83 ± 0.41 | 450 ± 110 | 38 ± 51 |
|  | surface (n = 13) | 12 ± 13 | 0.61 ± 0.25 | 480 ± 84 | 11 ± 6.1 |

Median concentrations (µM) ± IQR of salinity-corrected nutrients by sub-watershed and water type for samples collected during dry and wet seasons.
